# A Novel Signature of 23 Immunity-Related Gene Pairs Is Prognostic of Cutaneous Melanoma

**DOI:** 10.3389/fimmu.2020.576914

**Published:** 2020-10-19

**Authors:** Ya-Nan Xue, Yi-Nan Xue, Zheng-Cai Wang, Yong-Zhen Mo, Pin-Yan Wang, Wei-Qiang Tan

**Affiliations:** ^1^ Department of Plastic Surgery, Sir Run Run Shaw Hospital, Zhejiang University School of Medicine, Hangzhou, China; ^2^ Department of Biological Science, College of Chemistry and Life Sciences, Zhejiang Normal University, Jinhua, China; ^3^ Key Laboratory of Carcinogenesis and Cancer Invasion of the Chinese Ministry of Education, Cancer Research Institute, Central South University, Changsha, China; ^4^ Department of Neurosurgery, The Third Xiangya Hospital, Central South University, Changsha, China

**Keywords:** immune related gene pairs (IRGPs), cutaneous melanoma (CM), immune micro-environment, prognosis, immune check point

## Abstract

In this study, we aimed to identify an immune-related signature for predicting prognosis in cutaneous melanoma (CM). Sample data from The Cancer Genome Atlas (TCGA; n = 460) were used to develop a prognostic signature with 23 immune-related gene pairs (23 IRGPs) for CM. Patients were divided into high- and low-risk groups using the TCGA and validation datasets GSE65904 (n = 214), GSE59455 (n = 141), and GSE22153 (n = 79). The ability of the 23-IRGP signature to predict CM was precise, with the stratified high-risk groups showing a poor prognosis, and it had a significant predictive power when used for immune microenvironment and biological analyses. We subsequently established a novel promising prognostic model in CM to determine the association between the immune microenvironment and CM patient results. This approach may be used to discover signatures in other diseases while avoiding the technical biases associated with other platforms.

## Introduction

The incidence of cutaneous melanoma (CM) is increasing more rapidly than any other cancer, and accounts for about 132,000 new cases and 65,000 deaths worldwide annually (1). CM is the most lethal form of skin cancer and is a serious public health concern. The primary clinical feature of CM is early stage metastasis, which is one of the most significantly increasing cancers in the United States (2). Siegel et al. reported that in 2018, it had been 91,270 new cases and 9,320 deaths in the United States, owing to this disease (3).

As most diagnoses are made in the terminal stage, surgical results are often poor. At present, chemotherapy is the first line of treatment for CM (4), although many cases respond poorly to such regimens due to a high prevalence of adverse drug reactions and resistance to chemotherapeutic agents.

CM is associated with strong immunogenicity; thus, immunotherapy is a promising treatment alternative (5). Initial clinical trials using interferon-α (6) and high-dose interleukin-2 for advanced cases of CM (7) reported successful results. In addition, immune checkpoint inhibitors (ICIs), such monoclonal antibodies against cytotoxic T-lymphocyte-associated protein (CTLA-4) (8) and programmed cell death protein 1 (PD-1) (9), have provided meaningful results in the clinical outcomes against advanced melanoma, as demonstrated by improved survival and a greater curative effect for an increasing proportion of patients with CM.

However, despite broad efforts to identify novel prognostic biomarkers, the clinical behavior of CM remains unpredictable, rendering the prediction of survival time and tumor stage particularly difficult (10). Therefore, novel biomarkers and patient-tailored treatments are greatly needed, especially for patients at higher risk of recurrence. Although the immune system plays an essential role in the development and progression of CM (11), few immunity-related genes (IRGs) have been identified for use as tumor markers for prognosis. Nowadays, many recent CM investigations have several limitations such as studies only one or a few immunity-related biomarkers, small sample datasets, lack of follow-up validations or lack of detailed and comprehensive immunity-related studies. Moreover, many studies have reported that genetic mutations and the interactions between the tumor and its microenvironment can impact the biological features and malignant potential of CM. Considering that many immunity-related treatments have shown significant therapeutic effects, identification of the interactions between cancer cells and the host immune response is especially important (12, 13).

In this study, 23 IRGs associated with CM were identified from the whole transcriptome sequencing (RNA-seq) data retrieved from The Cancer Genome Atlas (TCGA) (14) and the ImmPort dataset (15). Then, three microarray datasets (GSE65904, GSE59455, and GSE22153) were selected from the Gene Expression Omnibus (GEO) database (16) to verify the usefulness of this 23 IRG pair (IRGP) signature for the prognosis of CM. Moreover, the possible relationships between clinical pathological factors and the prognostic signature were explored to validate the predictive efficacy and accuracy of the IRGP. Finally, analyses of immune cell infiltration, the tumor microenvironment, and biological functions of different risk groups based on the 23 IRGPs were performed.

## Materials and Methods

### CM Patient Data

In this retrospective study, four independent RNA-seq datasets and clinical data from diverse, high-throughput sequencing platforms were comprehensively analyzed. A CM dataset (n = 460) was downloaded from the TCGA data portal (https://portal.gdc.cancer.gov) and randomly assigned to a training dataset (n = 230) or a testing dataset (n = 230). In addition, the GSE65904 (n = 214), GSE59455 (n = 141), and GSE22153 (n = 79) datasets were downloaded from the GEO database (http://www.ncbi.nlm.nih.gov/geo/) for validation of the IRGP signature. In total, 905 samples were available for analysis. A file containing 1534 IRGs that was downloaded from the ImmPort database (https://immport.niaid.nih.gov) and the CIBERSORT analytical tool (https://cibersort.stanford.edu/) were used to determine an estimation of the abundances of 22 distinct cell types in a mixed cell population based on gene expression data. Immunohistochemical images of CM patients were downloaded from The Human Protein Atlas dataset (http://www.proteinatlas.org/). All data was available for free from website.

### Data Preprocessing

All data were preprocessed using R software (version 3.6.2). If more than one probe was matched to the same target gene, the average value of the probe was calculated to replace the expression level of the single gene. If there were multiple samples from the same patient, the average value of each gene was used as the expression level of that gene.

### Establishment of Prognostic IRGPs Based on the TCGA Dataset

The TCGA CM dataset was randomly divided into a training group and a testing group by R package “caret” with the ratio of training group samples to test group samples is 0.5. The distribution of CM patients gender (p = 0.068), age (p = 0.047), clinical stage (p = 0.036), follow-up time (p < 0.0001), death rate (p < 0.0001) and the number of CM samples in the dichotomies was similar between the two groups (17, 18). The GSE65904, GSE59455, and GSE2215 datasets were employed as the validation group. IRGs with relatively high variation (median absolute deviation >0.5) were extracted from the platforms, as described previously. For the pairwise comparison of a specific sample, two IRGs were paired off, and if the expression of the first IRG was higher than that of the second, the two IRGs were merged as an IRGP and assigned a score of 1; otherwise, a score of 0 was assigned. Then, IRGPs with score of 1 or 0 in over 80% of the specimens both in the training and testing groups were selected as potential prognostic IRGPs. Based on the results of a log-rank test, IRGPs with a false discovery rate (FDR) <0.001 (n = 23) were retained and entered into a least absolute shrinkage and selection operator (LASSO) penalized Cox regression model (iterations = 1000). The median value of the risk score was used as a cut-off to divide the patients into high- and low-risk groups. Next, a heat map, risk score map, state map of overall survival (OS), and 1-, 3-, and 5-year receiver operating characteristic (ROC) curves were created, and the concordance (C)-index was calculated. Then, the IRGPs were integrated with other clinical factors to construct a nomogram and a calibration curve. Finally, univariate and multivariate Cox regression analyses were performed to determine whether the 23 IRGPs were independent from other clinical parameters.

### Verification and Assessment of the IRGP Signature for the Prediction of OS

The TCGA testing dataset and three microarray data files were selected to validate the signature comprised of 23 IRGPs. Every dataset was stratified into high- and low-risk groups based on the cut-off value of the prognostic signature. Next, the log-rank test and Cox analysis were performed, and a graph of OS was created to calculate the C-index between the high- and low-risk groups in each dataset. Finally, the signature of the 23 IRGPs was compared to the 1-, 3-, and 5-year area under the ROC curves (AUCs) and the C-indices.

### Immune Cell Infiltration in CM

The CIBERSORT analytical tool (19) was used to explore the enrichment of immune cells in the two CM risk groups. CIBERSORT is a tool used for deconvolution of the expression matrix of immune cell subtypes based on the principle of linear support vector regression, which can estimate the enrichment of various immune cell types in CM. Based on the CIBERSORT results, a radar chart of significantly differentially expressed IRGs between the two risk groups was constructed. All procedures were performed using R software (version 3.6.2).

### Biological Function Analysis of the 23 IRGPs

Gene ontology (GO) and Kyoto Encyclopedia of Genes and Genomes (KEGG) pathways analyses of the two risk groups were performed using the R bioconductor package “fgsea.” GO analysis and KEGG pathways files (c5.all.v7.0.symbols.gmt and c2.cp.kegg.v7.1.symbols.gmt, respectively) were downloaded from the Gene Set Enrichment Analysis (GSEA) website (https://www.gsea-msigdb.org/gsea/datasets.jsp) (20). Gene sets with an FDR-adjusted probability (p) value <0.05 were considered statistically significant.

### Statistical Analysis

All statistical analyses were performed using the software packages R (version 3.6.2; www.r-project.org) and Prism 8 (GraphPad Software Inc., San Diego, CA, USA). Training group and testing group were randomly divided by R package “caret”. OS curves were plotted using the R packages “survival” and “survminer.” A heat map of the IRGPs, risk score map, and OS status graph were created using the R package “pheatmap.” A model of prognostic IRGPs was established using the R package “glmnet” (21). Univariate and multivariate Cox regression analyses were performed using the R package “survival.” ROC curves were constructed using the R package “survivalROC.” A nomogram and calibration curves were plotted using the R packages “rms,” “foreign,” and “survival.” The C-index was computed using the R package “Hmsic.” Immune cell infiltration was processed with the R packages “e701,” “limma,” and “fmsb” (22). The tumor environment plot, based on the R package “estimate” (23), and the expression levels of six single key genes were determine using the R package “ggpubr.” GO and KEGG analyses were conducted using the R package “fgsea.”

## Results

### Establishment, Definition, and Genetic Variation of the IRGP Signature

**Figure 1:**
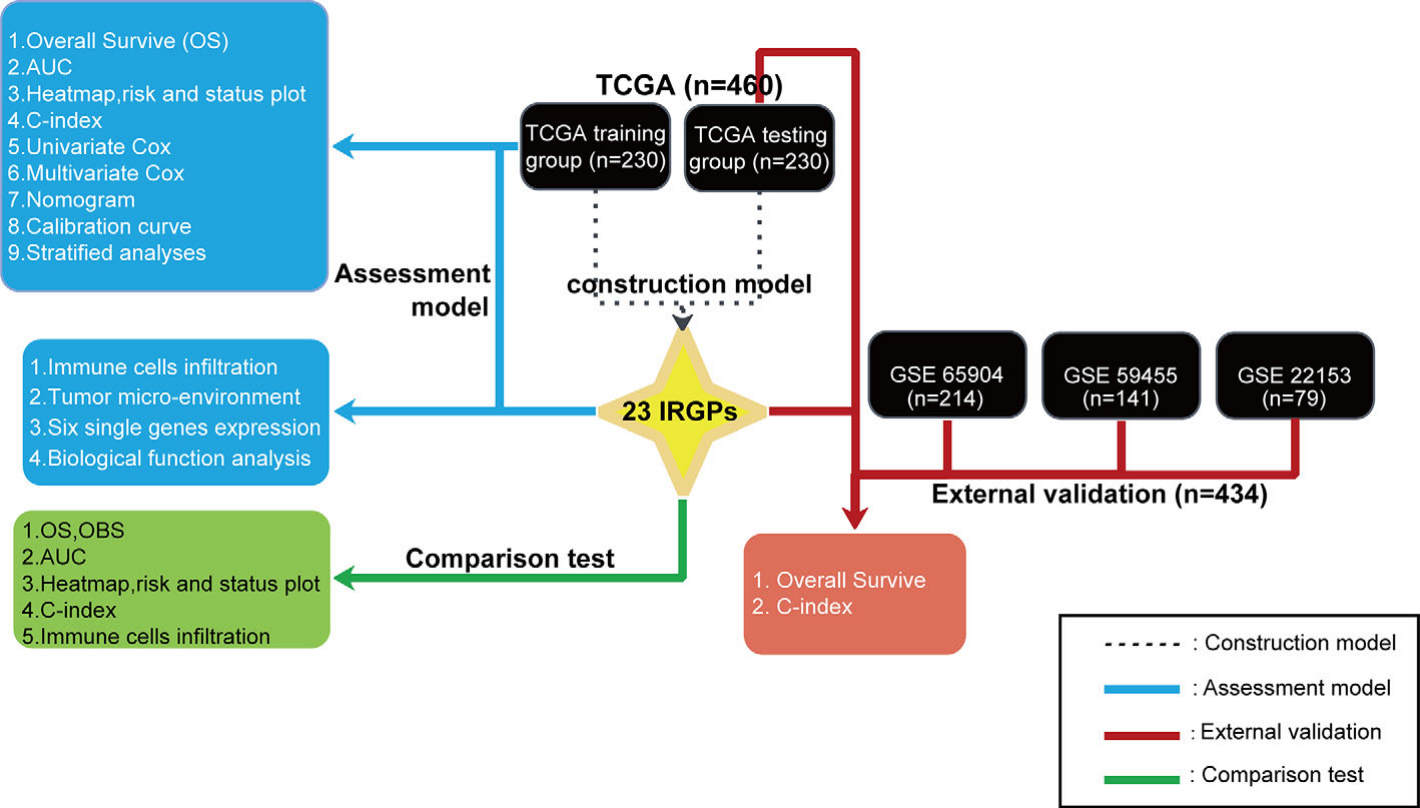
A flowchart of the establishment and validation of 23 IRGPs. The TCGA data were divided into a training cohort (230), which was applied to identify potential IRGPs, and a testing cohort (230). A validate cohort of the datasets GSE65904, GSE59455, and GSE22153 was used to verify the 23 IRGPs, which were also compared to the IRGP-OBS and IRGs.

**Figure 2:**
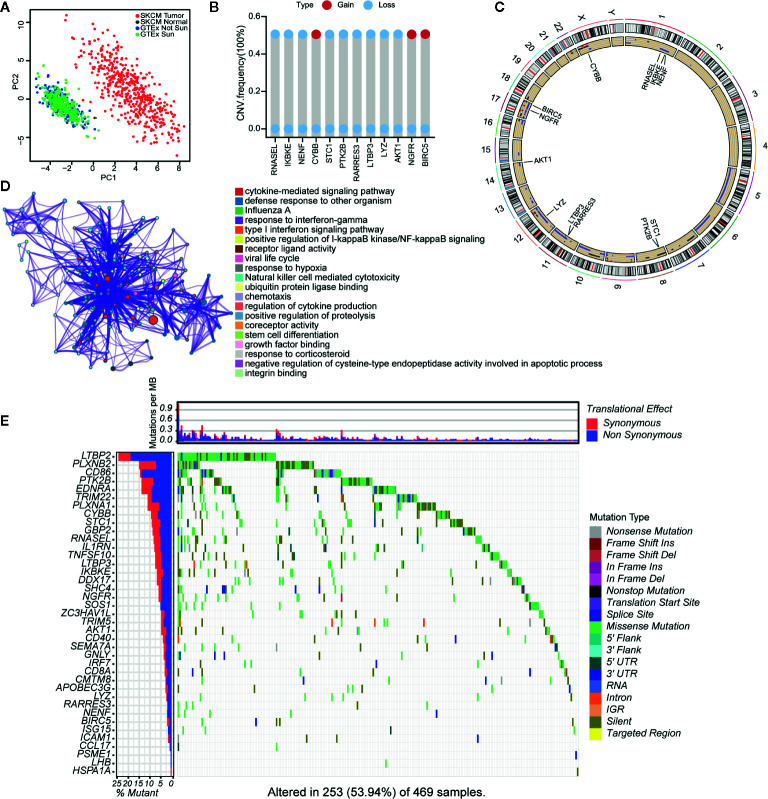
Landscape of genetic and expression variation of 39 IRGs in CM. **(A)** the Principal Component Analysis (PCA) of 39 unique IRGs to distinguish tumors and normal samples in TCGA cohort and GTEx normal skin cohort. Normal samples and tumor samples were identified without intersection, indicating the two subgroups were well distinguished based on the expression profiles of 39 IRGs. GTEx normal skin exposed sun were marked green, GTEx normal skin not exposed sun were marked blue, TCGA normal sample were marked gray and TCGA tumor samples were marked with red. **(B)** The CNV variation frequency of 12 IRGs in TCGA cohort. The blue dot meaning deletion frequency and the red dot meaning amplification frequency. The height of the column represented the alteration frequency. **(C)** The location of CNV alteration of 12 IRGs on 23 chromosomes using TCGA cohort. **(D)** The biological functional enrichment analysis and interaction network of enriched terms for 39 IRGs. **(E)** The mutation frequency of 39 IRGs in 460 patients with CM from TCGA mutation dataset. Each column represented individual patients. The number on the left showed the mutation frequency in each gene. The upper barplot showed the mutations per MB, Synonymous, red; Non Synonymous, blue. The right annotation list showed the different variant type.

**Figure 3:**
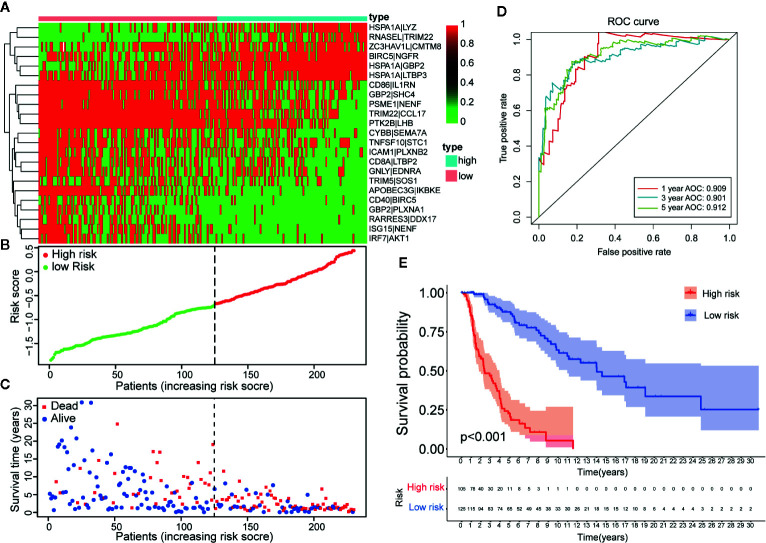
Establishment and assessment of a 23-IRGP signature. **(A)** A heat map of the risk scores of the 23 IRGPs. **(B)** According to the 23 IRGPs, the training cohort was divided into high and low immune risk groups. The red and green points represent the risk scores of the high and low risk groups, respectively. **(C)** A plot of OS based on the 23 IRGPs. The red points represent deaths, while the blue points represent survivors. **(D)** The AUCs for 1-, 3-, and 5-year OS in the training cohort were 0.909, 0.901, and 0.912, respectively. **(E)** According to the OS curve, OS was poorer for the high risk group as compared to the low risk group in the training cohort (p < 0.001).

A flowchart of the establishment and validation of the 23 IRGPs is presented in **Figure 1**. The TCGA dataset was divided into a training dataset (n = 230) and a testing dataset (n = 230) (**Table S2**). Filter analysis was applied to establish a prognostic model of 1,811 unique IRGs that were obtained from the ImmPort database (accessed on January 4, 2020). In total, 620 IRGs with a median absolute deviation >0.5 were common among the datasets. After removing any IRGPs with a score of 0 or 1 in <20% or >80% of the samples in the TCGA training and testing datasets, a total of 74,750 IRGPs remained. Of these, 6,800 prognostic IRGPs were significantly associated with OS (FDR < 0.001), as determined by log-rank testing. Finally, 23 IRGPs consisted of 39 IRGs were selected for the LASSO penalized Cox regression model (**Figure 3A**), including 39 unique IRGs, most of which encoded molecules related to antimicrobials and cytokines (**Table 1**). Furthermore, we conducted Principal Component Analysis (PCA), bioligical function analysis and genetic and expression variation of the 39 unique IRGs using GEPIA web (gepia.cancer-pku.cn/), Metascape (https://metascape.org/gp/index.html) and R package “RCircos” and “GenVisR”. We found that the genes expressed in TCGA tumor samples were independent parts, compared to TCGA normal sample (only one sample), GTEx normal skin exposed samples or not to the sun (**Figure 2A**). What’s more, **Figure 2D** showed 39 IRGs were positively correlated with cancer immune-related biological functions, including cytokine-mediated signaling pathway, defense response to other organism, Influenza A, response to interferon-gamma, and type I interferon signaling pathway. We first summarized the incidence of copy number variations and somatic mutations of 39 IRGs in CM. The investigation of CNV alteration frequency showed that only 12 IRGs had alteration, and most were focused on the deletion in copy number, while CYBB, NGFR and BIRC5 had a widespread frequency of CNV amplification (**Figure 2B**). The location of CNV alteration of 12 IRGs on chromosomes was shown in **Figure 2C**. Among 460 samples downloaded from TCGA-CM mutation dataset, 253 experienced mutations of 39 IRGs, with frequency 53.94%. We found that the LTBP2 exhibited the highest mutation frequency followed by PLXNB2, while almost antigen processing and presentation molecules (HSPA1A, ICAM1, and PSME1) as well as cytokines (CCL17 and LHB) did not show any mutations in CM samples (**Figure 2E**). Further analyses revealed a significant mutation co-occurrence relationship between IKBKE and CYBB, IKBKE and CD8A, GPB2 and CD8A, GNLY and CD40, LYZ and TNFSF10, along with GPB2 and PSME1 (**Figure S1**). To explore whether the above genetic variations influenced the expression of 39 IRGs in CM patients, we investigated the mRNA expression levels of genes between skin normal samples (GTEx skin normal sample and TCGA skin normal sample) and tumor samples, and found that the alterations of mutation could be the prominent impact factors resulting in perturbations on the 39 IRGs expression. Compared to normal skin tissues, 39 IRGs with high mutation obviously lower expression in CM samples (e.g., LTBP2, CD86, EDNRA, TRIM22, CYBB, STC1, GBP2, and RNASEL), and vice versa (e.g., LHB, PSME1, CCL17, ICAM1, ISG15, and BIRC5) (**Figure 2E** and **Figure S2**). The above analyses presented the highly heterogeneity of genetic and expressional alteration landscape in 39 IRGs between normal and CM tissues, indicating that the expression imbalance of 39 IRGs played a important role in the CM occurrence and progression.

**Table 1:** Information on the 23-IRGP.

Gene Pair1	Full Name	Immune Processes	Gene Pair2	Full Name	Immune Processes	Coefficient
CD8A	CD8a molecule	Antigen_Processing_and_Presentation, Antimicrobials, TCRsignalingPathway	LTBP2	latent transforming growth factor beta binding protein 2	Cytokines	−0.009639653
HSPA1A	heat shock 70kDa protein 1A	Antigen_Processing_and_Presentation	LYZ	lysozyme (renal amyloidosis)	Antimicrobials	0.098898359
HSPA1A	heat shock 70kDa protein 1A	Antigen_Processing_and_Presentation	GBP2	guanylate binding protein 2, interferon-inducible	Antimicrobials	0.06573857
HSPA1A	heat shock 70kDa protein 1A	Antigen_Processing_and_Presentation	LTBP3	latent transforming growth factor beta binding protein 3	Cytokines	0.00106882
ICAM1	intercellular adhesion molecule 1	Antigen_Processing_and_Presentation, NaturalKiller_Cell_Cytotoxicity	PLXNB2	plexin B2	Chemokine_Receptors, Cytokine_Receptors	-0.039340917
PSME1	proteasome (prosome, macropain) activator subunit 1 (PA28 alpha)	Antigen_Processing_and_Presentation	NENF	neuron derived neurotrophic factor	Cytokines	−0.008677831
ZC3HAV1L	zinc finger CCCH-type, antiviral 1-like	Antimicrobials	CMTM8	CKLF-like MARVEL transmembrane domain containing 8	Cytokines	0.037052249
APOBEC3G	apolipoprotein B mRNA editing enzyme, catalytic polypeptide-like 3G	Antimicrobials	IKBKE	inhibitor of kappa light polypeptide gene enhancer in B-cells, kinase epsilon	Antimicrobials	-0.511310011
CYBB	cytochrome b-245, beta polypeptide	Antimicrobials	SEMA7A	semaphorin 7A, GPI membrane anchor (John Milton Hagen blood group)	Chemokines, Cytokines	−0.0389128
TRIM5	tripartite motif-containing 5	Antimicrobials	SOS1	son of sevenless homolog 1 (Drosophila)	NaturalKiller_Cell_Cytotoxicity, TCRsignalingPathway	−0.064111635
TNFSF10	tumor necrosis factor (ligand) superfamily, member 10	Antimicrobials, Cytokines, NaturalKiller_Cell_Cytotoxicity, TNF_Family_Members	STC1	stanniocalcin 1	Cytokines	−0.089600117
RNASEL	ribonuclease L (2’,5’-oligoisoadenylate synthetase-dependent)	Antimicrobials	TRIM22	tripartite motif-containing 22	Antimicrobials	0.019575179
RARRES3	retinoic acid receptor responder (tazarotene induced) 3	Antimicrobials	DDX17	DEAD (Asp-Glu-Ala-Asp) box polypeptide 17	Antimicrobials	−0.036983795
CD40	CD40 molecule, TNF receptor superfamily member 5	Antimicrobials, Cytokine_Receptors	BIRC5	baculoviral IAP repeat-containing 5	Antimicrobials	−0.008823763
ISG15	ISG15 ubiquitin-like modifier	Antimicrobials	NENF	neuron derived neurotrophic factor	Cytokines	−0.225283963
GNLY	granulysin	Antimicrobials	EDNRA	endothelin receptor type A	Chemokine_Receptors, Cytokine_Receptors	−0.027334821
IRF7	interferon regulatory factor 7	Antimicrobials	AKT1	v-akt murine thymoma viral oncogene homolog 1	BCRSignalingPathway, TCRsignalingPathway	−0.035142572
TRIM22	tripartite motif-containing 22	Antimicrobials	CCL17	chemokine (C-C motif) ligand 17	Antimicrobials, Chemokines, Cytokines	−0.270054236
BIRC5	baculoviral IAP repeat-containing 5	Antimicrobials	NGFR	nerve growth factor receptor (TNFR superfamily, member 16)	Cytokine_Receptors	0.211653036
GBP2	guanylate binding protein 2, interferon-inducible	Antimicrobials	PLXNA1	plexin A1	Chemokine_Receptors, Cytokine_Receptors	−0.031264198
GBP2	guanylate binding protein 2, interferon-inducible	Antimicrobials	SHC4	SHC (Src homology 2 domain containing) family, member 4	NaturalKiller_Cell_Cytotoxicity	−0.09011139
PTK2B	PTK2B protein tyrosine kinase 2 beta	Antimicrobials, NaturalKiller_Cell_Cytotoxicity	LHB	luteinizing hormone beta polypeptide	Cytokines	−0.397220258
CD86	CD86 molecule	Antimicrobials	IL1RN	interleukin 1 receptor antagonist	Cytokines, Interleukins	−0.018374851

**Figure 4:**
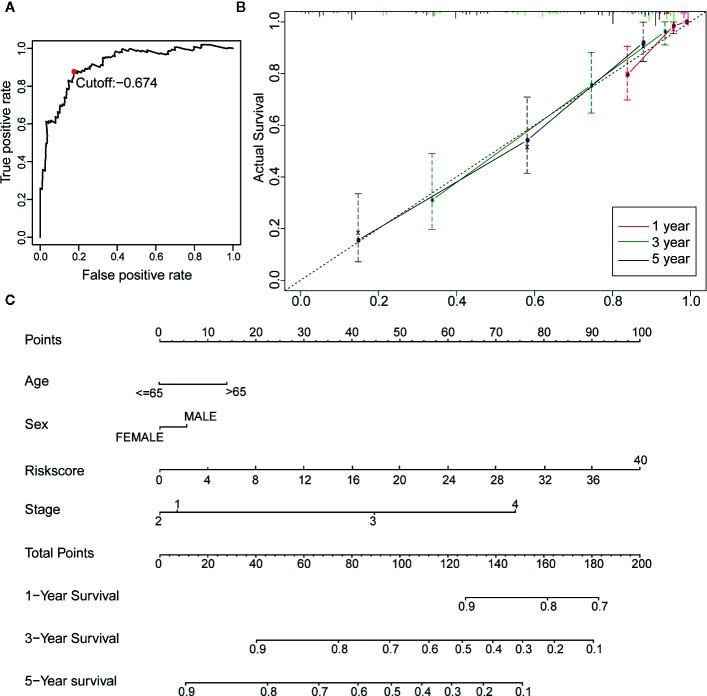
Construction of a Robust nomogram in TCGA training dataset. **(A)** A time-dependent ROC curve for IRGPs in the training and testing dataset. An IRGP score of −0.674 was used as a cut-off to assign patients to the high- or low-risk group. **(B)** The 1-, 3-, and 5-year calibration curves of the nomogram. **(C)** A nomogram of OS was established by 23-IRGP risk score and other clinicopathological parameters.

Twenty-three IRGPs were used to calculate a risk score to predict the 5-year OS rate of each patient in the training cohort. The analysis of the 5-year dependent ROC curve revealed that the best cut-off value of the 23 IRGPs to stratify patients in the training cohort and testing cohort into the high- or low-risk group was −0.674 (**Figure 4A**). These data suggested that the high-risk group had a higher risk index than the low-risk group. A higher risk score means a higher number of deaths (**Figures 3B, C**), indicating that OS was significantly poorer for the high-risk group than for the low-risk group (p < 0.001; **Figure 3E**). As shown in **Figure 3D**, the AUC values (24) for the 1-, 3-, and 5-year OS rates of the training cohort were 0.909, 0.901, and 0.912 (**Table S3**), respectively, and the C-index was 0.775 [95% confidence interval (CI) = 0.748–0.802] (**Figure 6E**). Moreover, the AUC values for the 1-, 3-, 5-year OS rates of the testing cohort and TCGA dataset was also shown in **Table S3**. A nomogram of OS was created by combining all of the clinicopathological factors, including age, sex, tumor stage, and the IRGP risk score, to predict the prognosis of CM (**Figure 4C**). The IRGP risk score made a major contribution to the nomogram, and the 1-, 3-, and 5-year calibration curves (**Figure 4B**) demonstrated the promising predictive ability of the nomogram, moreover, the nomograms of TCGA-test dataset and TCGA dataset were shown in **Figure S7**. The 1-, 3-, and 5-year calibration curves of TCGA-test dataset and TCGA dataset were shown in **Figures S9** and **S10**.

**Figure 5:**
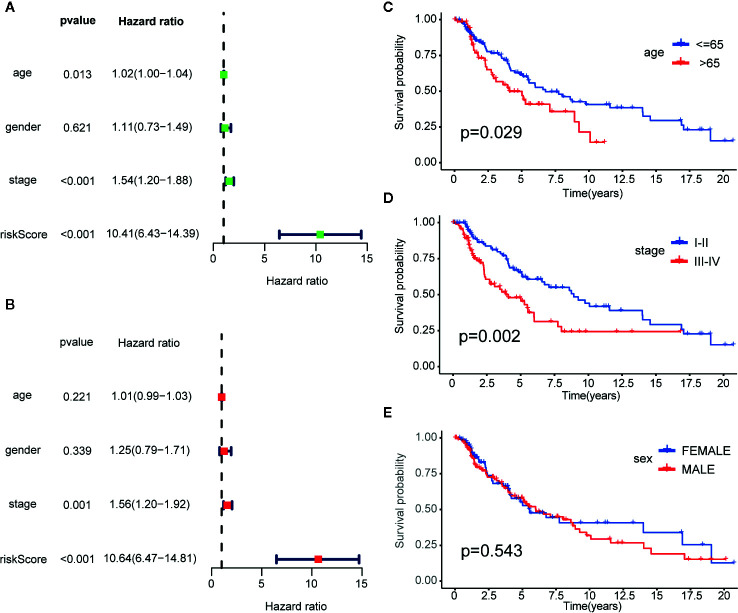
Cox proportional hazards model and stratified analysis of the training cohort revealed 23-IRGP was a independent prognostic factors. **(A)** Age, stage and risk score were the independent prognostic factors in Univariate Cox analysis. **(B)** Stage and risk score were the independent prognostic factors in Multivariate Cox analysis. Stratified analyses applied by age (p = 0.029) **(C)**, tumor stage (p = 0.002) **(D)** and sex (p = 0.543) **(E)** demonstrated the predictive of 23-IRGP in OS of CM patients.

Univariate and multivariate Cox proportional hazards regression analyses of the TCGA dataset were performed to further assess the prognostic accuracy of the IRGPs for other clinical elements. The results of the univariate and multivariate Cox analyses showed that the signature of the 23 IRGPs and clinical factors, such as tumor stage, were indeed predictive of prognosis. However, although the IRGP signature was highly predictive of prognosis, the p-value was notably low (**Figures 5A, B** and **Table 2**).

**Table 2:** Univariate Cox and Multivariate Cox analysis of clinicopathological factors and risk signatures.

Variable	Univariate Cox analysis of clinicopathologicalfactors and risk signatures			Multivariate Cox analysis of clinicopathologicalfactors and risk signatures		
**TCGA-train dataset**						
id	HR	95% CI	p-value	HR	95% CI	p-value
age	1.02	1.00–1.04	0.013	1.01	0.99–1.03	0.221
gender	1.11	0.73–1.49	0.621	1.25	0.79–1.71	0.339
stage	1.54	1.20–1.88	0.001	1.56	1.20–1.92	0.001
riskScore	10.41	6.43–14.39	1.49E-21	10.64	6.47–14.81	1.15E-20
TCGA-test dataset						
id	HR	95% CI	p-value	HR	95% CI	p-value
age	1.02	1.01–1.03	0.002	1.02	1.01–1.03	0.002
gender	0.94	0.60–1.28	0.805	0.84	0.53–1.15	0.469
stage	1.27	1.02–1.52	0.036	1.3	1.03–1.57	0.029
riskScore	2.12	1.44–2.80	0	2.08	1.41–2.75	0.0002
TCGA dataset						
id	HR	95% CI	p-value	HR	95% CI	p-value
age	1.02	1.01–1.03	0.011	1.01	0.98–1.04	0.010
gender	1.04	0.60–1.48	0.701	1.02	0.61–1.43	0.412
stage	1.42	1.31–1.63	0.006	1.13	0.83–1.43	0.008
riskScore	6.32	2.14–10.50	0.002	5.11	3.12–7.10	0.009
GSE65904 validation dataset						
id	HR	95% CI	p-value	HR	95% CI	p-value
age	0.99	0.98–1.01	0.567	1	0.99–1.02	0.592
gender	1.37	0.90–1.84	0.143	1.31	0.86–1.76	0.205
stage	2.35	1.53–3.17	8.55E-05	2.77	1.78–3.76	5.53E-06
riskScore	1.76	1.34–2.18	4.48E-05	1.98	1.49–2.47	1.87E-06
GSE59455 validation dataset						
id	HR	95% CI	p-value	HR	95% CI	p-value
age	0.99	0.98–1.00	0.177	0.99	0.98–1.00	0.112
gender	1.24	0.84–1.64	0.283	1.17	0.80–1.54	0.421
stage	0.77	0.67–0.87	0	0.76	0.66–0.86	8.68E-05
riskScore	1.35	0.78–1.92	0.141	1.64	0.94–2.34	0.042
GSE22153 validation dataset						
id	HR	95% CI	p-value	HR	95% CI	p-value
age	1.01	0.99–1.03	0.372	1.01	0.99–1.03	0.547
gender	0.99	0.55–1.43	0.967	1.32	0.71–1.93	0.378
stage	1.69	0.66–2.72	0.27	1.05	0.33–1.77	0.938
riskScore	1.95	1.15–2.75	0.013	2.07	1.16–2.98	0.014

Stratified analyses of patient age, tumor stage, and sex were also conducted. First, all patients in the TCGA training dataset were stratified by age into a young dataset (age ≤ 65 years) or an old dataset (age > 65 years), where OS was expected to be better for the younger patients (p = 0.029, **Figure 5C**). Then, all patients from the TCGA training dataset were further divided into an early onset dataset (stages I and II) or a later onset dataset (stages III and IV). Similar results were observed for the late dataset (p = 0.002, **Figure 5D**). Finally, all patients were stratified by sex into a male dataset or a female dataset. As shown in **Figure 5E**, there was little difference in the OS rate between males and females (p = 0.543), possibly due to the small number of samples.

Collectively, the results indicate that the predictive ability of the 23-IRGP signature was independent of other clinical parameters and was predictive of OS of CM patients.

### Verification of Ability of the 23-IRGP Signature to Predict OS

**Figure 6:**
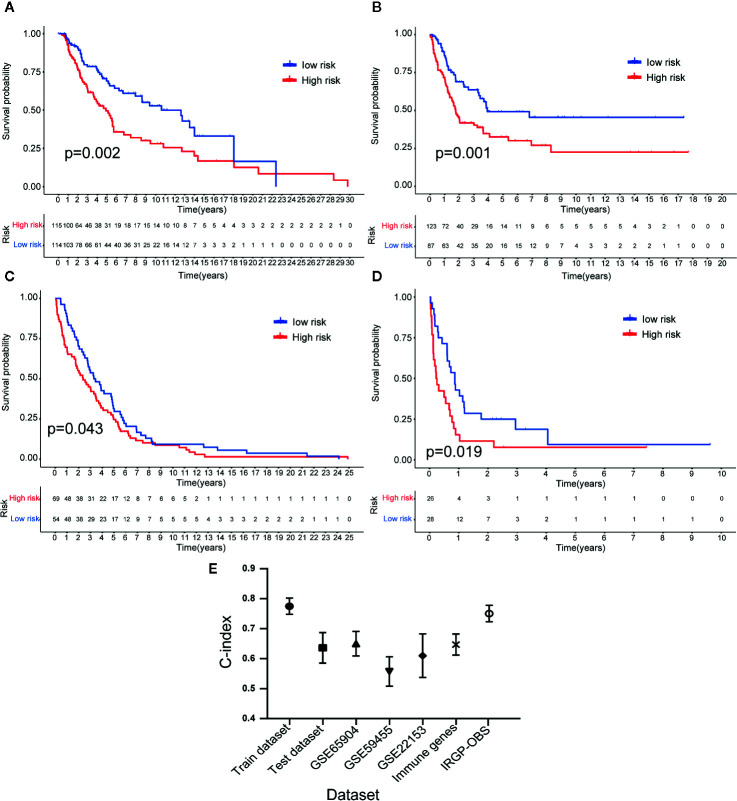
Validation of the 23-IRGP signature. As indicated, OS was poorer for the high-risk group than the low-risk group of the testing cohort **(A)**. Datasets GES65904 **(B)**, GSE59455 **(C)**, and GSE54467 **(D)**. These results showed that the 23-IRGP signature had good predictive ability (p < 0.05) **(E)**. The C-index values for the TCGA training, testing cohorts and TCGA dataset, as well as the datasets GES65904, GSE59455, GSE54467, IRGs and IRGP-OBS were 0.775 (95% CI = 0.748–0.802), 0.636 (95% CI = 0.585–0.687), 0.691 (95% CI = 0.653–0.729), 0.650 (95% CI = 0.609–0.691), 0.557 (95% CI = 0.508–0.606), 0.610 (95% CI = 0.537–0.683), 0.647 (95% CI = 0.612–0.682), 0.751 (95% CI = 0.716–0.786), respectively.

To determine whether the 23-IRGP signature had consistent prognostic value in different risk groups, the validation datasets GES65904 (n = 214), GSE59455 (n = 141), and GSE54467 (n = 79) were applied for external validation. The risk score of each patient was calculated using the same 23-IRGP prognostic signature. Then, based on the median risk score, the patients were assigned to the low- or high-risk group. Interestingly, OS was poorer in the high-risk group (**Figures 6A–D**). The results of multivariate Cox regression analysis (**Table 2**) showed that, after adjustment for age, sex, and tumor stage (age is a continuous variable), the risk score from the 23-IRGP signature was an independent prognostic factor in the testing dataset [hazard ratio (HR) = 2.08, 95% CI = 1.41–2.75, p = 0.0002] and TCGA dataset (HR = 2.08, 95% CI = 1.41–2.75, p = 0.0002), as well as the GSE datasets [GSE65904 (HR = 5.11, 95% CI = 3.12–7.10, p = 0.009), GSE59455 (HR = 1.64, 95% CI = 0.94–2.34, p = 0.042), and GSE22153 (HR = 2.07, 95% CI = 1.16–2.98, p = 0.014)]. Finally, the C-index values for the TCGA training dataset, TCGA test dataset, TCGA dataset, GSE65904, GSE59455, and GSE22153 datasets were 0.775 (95% CI = 0.748–0.802), 0.636 (95% CI = 0.585–0.687), 0.650 (95% CI = 0.609–0.691), 0.691 (95% CI = 0.653–0.729), 0.557 (95% CI = 0.508–0.606), and 0.610 (95% CI = 0.537–0.683), respectively (**Figure 6E**), the AUC values for the 1-, 3-, 5-year OS rates of the these datasets were also shown in **Table S3**. Moreover, the nomograms and the 1-, 3-, and 5-year calibration curves of three GSE validation datasets were shown in **Figures S8**, and **S11–S13**.

### Immune Cell Infiltration, the Tumor Microenvironment, Potential of 23-IRGP as an Indicator of Response to Immunotherapy, and Analysis of Six Key Genes

**Figure 7:**
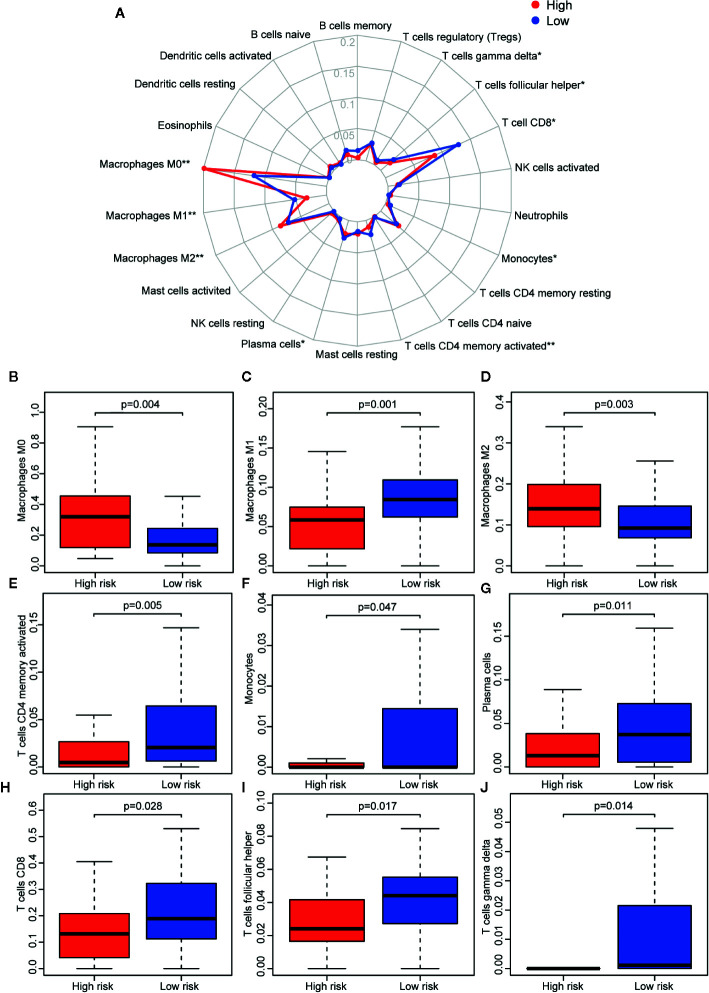
Immune infiltration status of the 23 IRGPs. **(A)** Summary of the abundances of 22 types of immune cells, as estimated with the use of the CIBERSORT analytical tool for different risk groups. **(B–J)** The abundance distribution of specific immune cells within different risk groups. The abundances of M0 macrophages (p = 0.004) and M2 macrophages (p = 0.003) were significantly greater in the high risk group, while the abundances of M1 macrophages (p = 0.001), activated CD4+ memory T cells (p = 0.005), monocytes (p = 0.047), plasma cells (p = 0.011), CD8+ T cells (p = 0.028), follicular helper T cells (p = 0.017), and gamma delta T cells (p = 0.014) were significantly enriched in the low risk group. *p < 0.05, **p < 0.01 (t-test).

**Figure 8:**
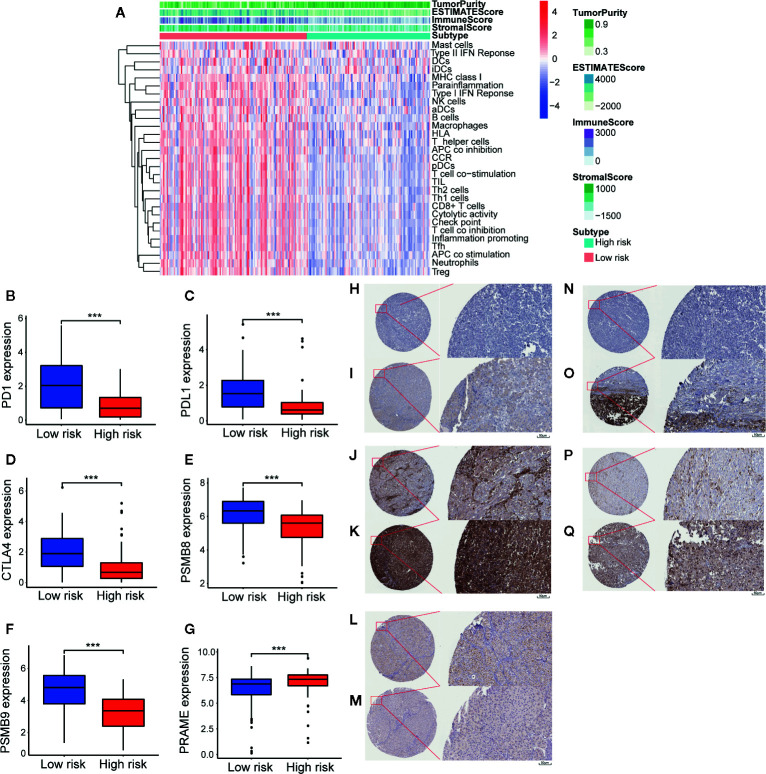
Tumor micro-environment (TME) and key genes expression in two different groups. In TME, “TumorPurity” is the percentage of tumor cells, “ImmuneScore” is the percentage of Immune cells, “StromalScore” is the percentage of stromal cells, “EstimateScore” is the percentage that merge the “ImmuneScore” and “StromalScore”. as we can see, high risk group had higher tumor purity with lower immune cells and stromal cells infiltration **(A)**. In low immune risk group, PD-1, PD-L1, CTLA-4, PSMB8, and PSMB9 were highly expressed **(B–F)**. PRAME were significant expressed in high risk group **(G)**. **(H)** IHC result of PD-1 protein in high risk group. Staining, not detected; intensity, negative; quantity, none; location, none. **(I)** IHC result of PD-1 protein in low risk group. Staining, low; intensity, weak; quantity, 75%–25%; location, cytoplasmic/membranous. **(J)** IHC result of PD-L1 protein in high risk group. Staining, not detected; intensity, negative; quantity, none; location, none. **(K)** IHC result of PD-L1 protein in low risk group. Staining, high; intensity, Strong; quantity, >75%; location, cytoplasmic/membranous. **(L)** IHC result of PSMB8 protein in high risk group. Staining, low; intensity, moderate; quantity, <25%; location, cytoplasmic/membranous nuclear. **(M)** IHC result of PSMB8 protein in low risk group. Staining, high; intensity, Strong; quantity, >75%; location, cytoplasmic/membranous nuclear. **(N)** IHC result of PSMB9 protein in high risk group. Staining, not detected; intensity, negative; quantity, none; location, none. **(O)** IHC result of PSMB9 protein in low risk group. Staining, medium; intensity, moderate; quantity, >75%; location, cytoplasmic/membranous nuclear. **(P)** IHC result of PRAME protein in high risk group. Staining, medium; intensity, moderate; quantity, 75%–25%; location, cytoplasmic/membranous. **(Q)** IHC result of PRAME protein in low risk group. Staining, not detected; intensity, weak; quantity, <25%; location, cytoplasmic/membranous. ***p < 0.01 (t-test).

Reportedly, the infiltration of immune cells is associated with the prognosis of CM patients. The CIBERSORT analytical tool can be used to estimate the abundances of immune cell subsets and has been used in many previous studies of the cancer microenvironment. Therefore, the CIBERSORT analytical tool was applied to estimate the relative abundances of 22 different immune cells for each patient. A comparative summary of the CIBERSORT output resulting from the two risk groups is shown in **Figure 7A**. Immune cells, such as M0, M1, and M2 macrophages; plasma cells; activated CD4+ memory T cells; monocytes; CD8+ T cells; follicular helper T cells; and gamma delta T cells, were enriched in the risk groups. As shown in **Figures 7B, D**, M0 macrophages (p = 0.004) and M2 macrophages (p = 0.003) were significantly high in the high-risk group, while the abundances of M1 macrophages (p = 0.001), activated CD4+ memory T cells (p = 0.005), monocytes (p = 0.047), plasma cells (p = 0.011), CD8+ T cells (p = 0.028), follicular helper T cells (p = 0.017), and gamma delta T cells (p = 0.014) were significantly enriched in the low-risk group (**Figures 7C, E–J**). Then, we estimated the tumor microenvironment (TME) in the two groups and found that the high-risk group had a higher tumor purity with lower immune cells and stromal cell infiltration (**Figure 8A**). Furthermore, as 23-IRGP had a potential of indicator of response to CM immunotherapy, the relationship between the 23-IRGP and ICIs, namely PD-1, PD-L1, and CTLA-4, were investigated. As shown in the **Figure S14**, the 23-IRGP was markedly negatively related with PD-1 and PD-L1 (rho = 0.321 and p < 0.001 for PD-1, and rho = 0.203 and p < 0.001 for PD-L1) (**Figures S14A, B**), and positively correlated with CTLA-4 (rho = 0.085 and p = 0.145, without statistical significance). Moreover, three ICIs were found to be highly expressed in the low-risk group of 23-IRGP prognosis signature (**Figures 8B–D**), and this result indicated that patients with low risk presented obviously higher expression levels of immune checkpoint genes (PD-1, PD-L1) compared with those in the high risk group (p < 0.001 for PD-1, and p < 0.001 for PD-L1) (**Figures 8B, C**), which demonstrated that the PD-1 and PD-L1 are involved in better immunotherapy efficacy, and their high expression is associated with better prognosis. The effect of cross-talk between 23-IRGP and ICIs on CM patients’ survival was shown in **Figures S14D–F**. According to the Meng Zhou et al. (25), we divided TCGA-CM patients into four clusters according to the connection of 23-IRGP and ICIs, and survival comparisons of three ICIs were presented among the four clusters. In PD-1 and PD-L1, Survival rate results showed that the 23-IRGP could significantly differentiate the result of patients with the same or similar levels of PD-1 and PD-L1 (P < 0.001, log- rank test) (**Figures S14D, E**). Relative to other three clusters, patients who had low 23-IRGP value with high level of PD-1 or PD-L1 were likely to have best survival results. However, patients who had high 23-IRGP value with low level of PD-1 or PD-L1 expression tended to the poorest consequence compared with the other three clusters (**Figures S14D, E**). Meanwhile, patients who had low level PD-1 or PD-L1 with low 23-IRGP value had better survival outcomes than the patients that had low PD-1 or PD-L1 with high 23-IRGP value. Furthermore, no obvious statistical significance was identified between the expression level of CTLA-4 and survival results for patients with 23-IRGP (P = 0.063, log- rank test) (**Figure S14F**). Collectively, these investigations between the 23-IRGP and ICIs made us to speculate that the 23-IRGP may have a predictive ability of the response to CM immunotherapy. Kalaora et al. reported that that the among melanoma patients, overexpression of PSMB8 and PSMB9 was predictive for better survival and improved response to immune-checkpoint inhibitors (26), and these genes were highly expressed in the low-risk group (**Figures 8E, F**). Interestingly, the PRAME gene, which is an independent biomarker of uveal melanoma metastasis (27), was also significant expressed in the high-risk group (**Figure 8G**). These results correlated with the immunohistochemical results downloaded from The Human Protein Atlas dataset (THPA) (**Figures 8H–Q**), which showed no results for CTLA-4, while the other five genes were expressed in melanoma tissue. Furthermore, in GEPIA, patients with high PD-1, PD-L1, CTLA-4, PSMB8, and PSMB9 expression showed better OS (**Figures S3A–E**). cBioPortal (https://www.cbioportal.org/) was used to determine the mutation rates of the different genes. According to the results, the probability of mutation for PD-1, PD-L1, CTLA-4, PSMB8, and PSMB9 were 5%, 1.9%, 1.6%, 5%, and 4% (**Figure S4A**), respectively. Poor OS was observed in cases where these genes were mutated (**Figure S4B**). In addition, in the GEPIA analysis, PRAME had no significant effect on OS (**Figure S3F**). However, further study will be needed to verify this result.

### Biological Function Analysis in the High-Risk Group Stratified by the 23-IRGP Signature

First, GSEA was used to investigate the results of the GO and KEGG pathway analyses between the high- and low-risk groups using genes that were more highly expressed in the high-risk group than the low-risk group. According to the GO analysis results, these genes were positively correlated with skin-related biological functions, including keratinization, epidermal cell differentiation, keratin filament, intermediate filament cytoskeleton, and skin development (padj < 0.05). A bubble graph of the 16 GO terms enriched in the high-risk group with a padj value < 0.05 is presented in **Figure S5**. Information on every GO term is provided in **Table S1**. As shown in **Figure S6**, several melanoma progression-related pathways, including oxidative phosphorylation (28, 29), retinol metabolism (30–32), and ribosome (33, 34), were significantly upregulated in the high-risk group (padj < 0.05). Collectively, the results obtained using the IRGP signature provide evidence of the molecular mechanisms affected by CM and, thus, the predictive power of this signature for the prognosis of CM patients.

### Comparison of IRGP Signature Model and Others in CM

**Figure 9:**
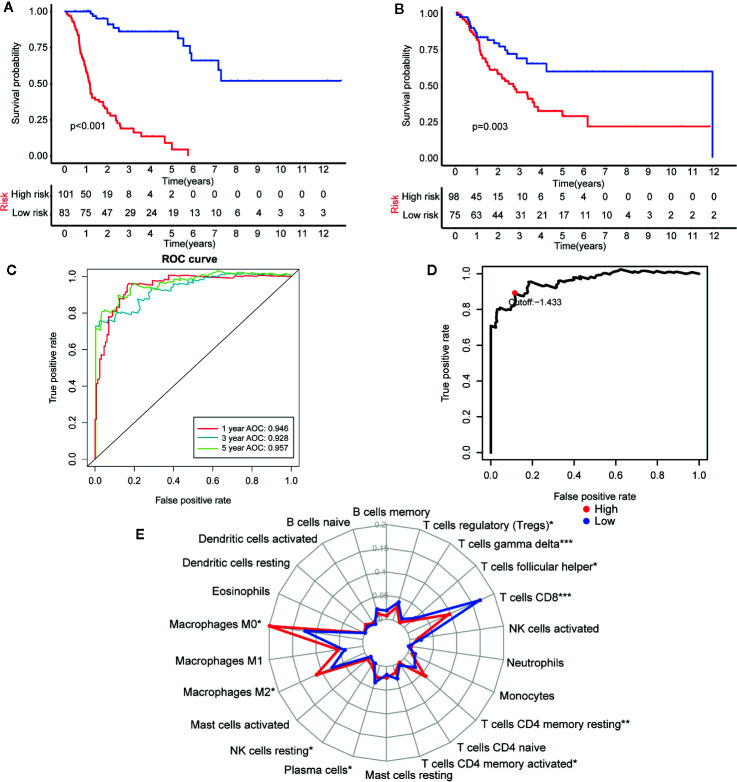
Establishment and assessment of the IRGP-OBS. **(A)** According to the OBS curve, OBS was poorer for the high risk group as compared to the low risk group in the training cohort (p < 0.001). **(B)** According to the OBS curve, OBS was poorer for the high risk group as compared to the low risk group in the training cohort (p = 0.003). **(C)** The AUCs for 1-, 3-, and 5-year OS in the training cohort were 0.946, 0.928, and 0.957, respectively. **(D)** A time-dependent ROC curve for IRGP-OBS in the training and testing dataset. An IRGP score of −1.433 was used as a cut-off to assign patients to the high- or low-risk group. **(E)** The abundances of M0 macrophages (p = 0.013), M2 macrophages (p = 0.049), T cells CD4 memory resting (p = 0.001) and NK cells resting (p = 0.035) were significantly greater in the high risk group, while the abundances of CD8+ T cells (p < 0.001), plasma cells (p = 0.043), follicular helper T cells (p = 0.025), gamma delta T cells (p < 0.001), T cells regulatory (p = 0.019), T cells CD4 memory activated (p = 0.011) were significantly enriched in the low risk group. *p < 0.05, **p < 0.01, ***p < 0.001 (t-test).

TCGA-CM dataset includes primary and metastatic samples, the primary samples submitted to sequence were initially diagnosed melanoma samples; however, the metastatic samples for sequencing were always from follow-up patients instead of initially diagnosed samples. So the TCGA-CM dataset did not always adopt the initially diagnosed melanoma samples for sequencing. So we established another prognostic model in CM determine the effectiveness of this approach according to the observed survival interval (OBS), which could be defined as the time interval from TCGA sampling to patient death or last follow-up (35). As is shown in **Figures 9A, B**, IRGP-OBS prognostic signature divided the TCGA training dataset and testing dataset into a low- or high-risk OBS group, respectively, with cut off value −1.433 (**Figure 9D**). Both in training and testing groups, high-risk group had a poor OBS than low-risk group. As shown in **Figure 9C**, the AUC values for the 1-, 3-, and 5-year OS rates of the training cohort were 0.946, 0.928, and 0.957, respectively, and the C-index was 0.751 (95% CI = 0.716–0.786) (**Figure 6E**). It is worth noting that, group’s immune cells infiltration of IRGP-OBS had a similar trend with 23-IRPG’s (**Figure 9E**). In this study, both IRGP prognostic and 23-IRGP prognostic models had predictive power of immune cells infiltration, with high AUC value and C-index value.

**Figure 10:**
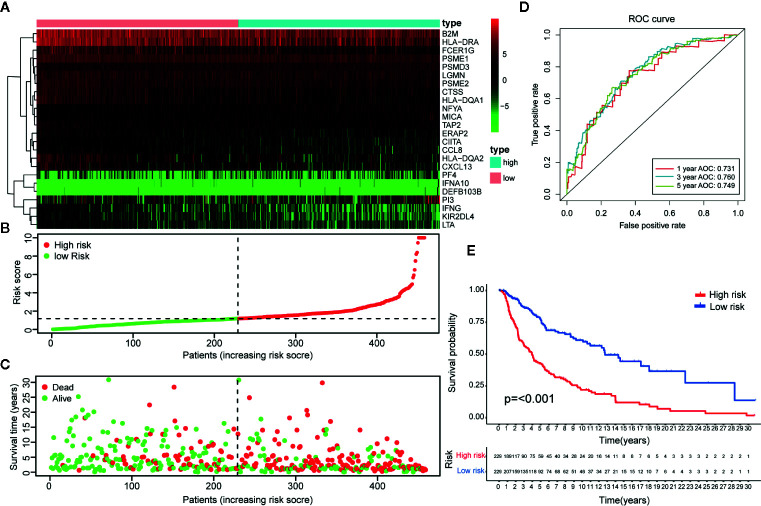
Identification of IRGs and comparison with IRGPs. **(A)** A heat map of the patient risk scores of the 24 IRGs. **(B)** Based on the 24 IRGs, patients in the TCGA dataset were assigned to the high- or low-immune risk groups. The red and green points represent the risk scores of the high and low risk groups, respectively. **(C)** A plot of OS based on the 24 IRGs. The red points represent deaths and blue points represent survivors. **(D)** The AUCs for 1-, 3-, and 5-year OS in the training cohort were 0.731, 0.760, and 0.749, respectively. **(E)** According to the OS curve, OS was poorer for the high risk group as compared to the low risk group in the training cohort (p < 0.001). These results showed that the prognostic signature of the IRGs had good predictive power, but with a smaller AUC and lower C-index (0.610 in **Figure 5E**) than the 23 IRGPs signature. Therefore, the 23-IRGP was more precisely predictive for CM.

The 23-IRGP prognostic signature was also compared with the prognostic signatures of individual IRGs. First, as the TCGA CM data had only one normal sample, the samples from the Genotype-Tissue Expression dataset (36) and TCGA CM dataset were merged. Then, significant differentially expressed IRGs were selected. Next, the LASSO penalized Cox regression model was applied to the TCGA clinical dataset, and 24 prognostic IRGs were selected for the final risk scoring model (**Figure 10A**). Most of the 24 prognostic IRGs coded for molecules related to antimicrobials and cytokines. The IRGs significantly stratified the TCGA dataset patients into a low- or high-risk OS group. These data suggested that the high-risk group had a higher risk index than the low-risk group, as a higher risk score was associated with a higher risk of death (**Figures 10B, C**). Moreover, the high-risk group had a significantly poorer OS than the low-risk group (p < 0.001) (**Figure 10E**). As shown in **Figure 10D**, the AUC values of the 1-, 3-, and 5-year OS rates were 0.731, 0.760, and 0.749, respectively, and the C-index was 0.647 (95% CI = 0.612–0.682) (**Figure 6E**). Collectively, these results demonstrate that the prognostic signatures of these IRGs had predictive ability but with a smaller AUC and lower C-index than the 23-IRGP signature, demonstrating that the 23-IRGP signature was the more precise predictive model in CM.

## Discussion

CM is a solid malignant tumor with strong immunogenicity with a rapidly increasing incidence rate worldwide. Since the approval of interferon-α for the treatment of CM in 1995, the potential of other immunotherapies have received much attention from researchers (37). Like with many other tumors, immune checkpoint (PD-1/PD-L1 and CTLA-4) blockade therapy has also become a target clinical. In 2011, ipilimumab, which targets CTLA-4, provided a major breakthrough in the clinical treatment of CM and was subsequently approved for marketing by the Food and Drug Administration (FDA). Ipilimumab is the first antibody drug to prolong the OS of patients with metastatic cancer (38). However, ipilimumab has been associated with some toxicity; thus, other immune surveillance sites have since been investigated, which has led to phase III clinical studies of the anti-PD-1 antibody drugs pembrolizumab and nivolumab. These drugs were approved for use by the FDA in September and December 2014, respectively. With lower anti-drug resistance and higher clinical safety, these anti-PD-1 antibody drugs offer hope to patients with advanced unresectable or metastatic CM (39, 40). Given that the results of single antibody drugs are limited and the many links between the occurrence and development of CM, a multiple-immune therapy strategy may have more prospects (41). It is, therefore, necessary to develop a prognostic signature using IRGs.

Due to the technical bias of sequencing platforms, gene expression data must be preprocessed for standardization, which is particularly significant when establishing prognostic signatures. To achieve robust prognosis prediction without the technical bias associated with different platforms, prognostic signatures of IRGPs can be established by pairwise comparison, which does not require preprocessing for data standardization. IRGP scores are calculated based on the expression levels of IRGs in the same sample. As such, the prognostic signature is not only able to overcome the batch effects of different platforms, but also does not require the scaling and normalization of data. In CM prognostic model, based on the AUC and C-index values, the prediction capability of IRGPs is more promising when compared with prognostic checkpoint (AUC = 0.729) (42), prognostic DNA methylation (AUC = 0.822) (43), prognostic IRGs that require preprocessing for data standardization. Moreover, this approach has been reported to be robust in other cancer-related studies (44, 45).

Given that the results of TCGA-CM dataset is made up of primary and metastatic samples, and the metastatic samples for sequencing were always from follow-up patients instead of initially diagnosed samples, IRGP-OBS prognostic model were established to make a predict comparison with 23-IRGP. As we expected, both IRGP-OBS model and 23-IRGP model had precious prediction capability with high AUC and C-index values. In addition, many researchers of CM also regarded OS as golden standard to evaluate the model predict power (43, 46), and there is no significantly different trend in immune cell infiltration in our study. Collectively, IRGP model could be well applied to survival probability and immune cell infiltration of CM patients, providing reference for immunotherapy.

In the present study, an IRGP signature was established using a LASSO penalized Cox regression model to predict OS in CM patients. The prognostic signature of the 23 IRGPs consisted of 46 unique IRGs. Most of the genes in the immune signature encoded molecules related to antimicrobials and cytokines, which play important roles in the response to stimuli and the immune microenvironment. Many of these IRGs have be shown to be related to cancer development and prognosis, expression of serine/threonine kinase 1 promotes melanoma metastasis (47), serum levels of C-C motif chemokine ligand 17 (CCL17) are an independent prognostic marker of distant metastasis of melanoma, and patients with 43% of patients with high CCL17 levels survived to 3 years (48). Singh et al. found that activation of intratumoral cluster of differentiation 40 induced T cell-mediated eradication of melanoma in the brain (49). Smith et al. discovered that endothelin 1 was enhanced in treated melanomas and conferred drug resistance *via* endothelin receptor type A (50). Ribonuclease L has been reported to interact with microRNA-146a as a sex-specific factor in melanoma (51), and semaphorin 7A has been found to reduce the pulmonary metastasis of melanoma (52).

In this study, according to 23-IRGP signature, ICIs, including PD-1, PD-L1, and CTLA-4, were highly expressed in the low-risk group which had better survival. When exploring the relationship between PD-1/PD-L1 and the 23-IRGP value, the 23-IRGP signature presented significant relationship with PD-1/PD-L1 expression. Moreover, the interaction between ICIs and the 23-IRGP indicated a combined prognostic effect on patient survival. M2 macrophages have been shown to promote growth and are related to poorer OS in melanoma patients, while M1 macrophages support tumor destruction and antigen presentation (53). Yamaguchi et al. found that anti-PD-1 antibody (nivolumab) therapy increased the activated effector memory phenotypes of central memory T cells and subsets of CD4+ and CD8+ central memory T cells, as well as Th1 plus T-helper follicular 1 cells (54), indicating that these immune cells can prolong patient survival when they are activated. The results of the present study revealed a significant increase in the abundance of infiltrating M0 and M2 macrophages in the high-risk immune group, while the abundances of infiltrating M1 macrophages, activated memory CD4+ T cells, CD8+ T cells, follicular helper T cells, monocytes, plasma cells, and gamma delta T cells were greater in the low-risk immune group, which was found to have a better survival rate. In addition, both the mRNA analysis and immunohistochemistry results showed that the high-risk group had higher tumor purity and lower infiltration of immune cells and stromal cells. Meanwhile, low-risk group had higher immune checkpoint inhibitors indicating that patients in the low-risk group may have better outcomes with immunotherapy. Nowadays, ICIs provide a new way to cacer immunotherapy, when exploring the relationship 23-IRGP signature and ICIs, 23-IRGP signature showed closely connections with ICIs expression. Moreover, the relationship between 23-IRGP and ICIs indicated an integrated prognostic power on patient OS, which is associated with previous result that M1 macrophages, activated CD4+ memory T cells, monocytes, plasma cells, CD8+ T cells, follicular helper T cells, and gamma delta T cells infiltration and ICIs expression may make a difference on patients’ OS and patients’ immunotherapy effect. Collectively, it may suggested that the 23-IRGP may have a predictive ability of the response to CM immunotherapy (25).

In our previous study, overexpression of PSMB8 and PSMB9 was found to be predictive of better survival and improved response to immune-checkpoint inhibitors. This was reflected in the low-risk group in the present study, in which PSMB8 and PSMB9 were highly expressed, indicating that the low-risk group had better survival rate and immunotherapy effect. Moreover, this indicates that a mutation in these genes will result in poor OS. Unexpectedly, PRAME, which acts as an independent biomarker in uveal melanoma metastasis, was also significantly expressed in the high-risk group, indicating that PRAME may also be a biomarker in CM. However, further study will be needed to verify these results. Thus, our research outcomes were closely in line with those of previous studies, demonstrating the precise predictive power of our platform (55).

The 23-IRGP signature identified three pathways (i.e., oxidative phosphorylation, retinol metabolism, and ribosome) that were highly related to the invasiveness of melanoma, suggesting that a high-risk score was correlated with increased melanoma metastasis and poorer survival. These results indicated the capability of the IRGP signature for predicting tumor invasion in CM patients.

Nevertheless, there were some limitations to this study that should be addressed. First, the 23-IRGP prognostic signature was based on a retrospective study using the TCGA CM dataset and validated using three microarray datasets from the GEO dataset. Thus, these results should be validated against other datasets with different sample attributes in a prospective cohort. Second, as the 23 IRGPs were used to construct a prognostic signature model, different prognostic signature models are needed for comparison. Third, further validation of the 23 IRGPs by quantitative real-time polymerase chain reaction, western blotting, and immunohistochemical analyses will be needed before this approach can be applied clinically. Fourth, due to the fact that the relevant CM data (such as patients that received PD-1, PD-L1, and CTLA-4 treatment) cannot be obtained, the analysis of cross-talk between the signature and ICIs cannot be compensated systematically at present.

## Conclusions

In conclusion, our findings indicate that our prognostic signature established using 23 IRGPs is a novel, promising model for predicting the prognosis of CM, indicating an association between the immune microenvironment and CM. This approach can be used to discover signatures in other diseases without the technical bias associated with different platforms.

## Data Availability Statement

Publicly available datasets were analyzed in this study. This data can be found here: TCGA dataset (https://www.cancer.gov/about-nci/organization/ccg/research/structural-genomics/tcga); GEO dataset (https://www.ncbi.nlm.nih.gov/gds/): GSE65904, GSE59455, and GSE22153.

## Author Contributions

Ya-NX conceptualized the project, all data analysis and wrote the first draft of the manuscript. Yi-NX, Z-CW, Y-ZM, and P-YW contributed to processing, analysis, and interpretation of the data. W-QT contributed to guide the data analysis, and manuscript writing. All authors contributed to the article and approved the submitted version.

## Funding

This study was supported by the National Natural Science Foundation of China (No. 81671918), the National Key Research Program of China (No. 2016YFC1101004), and Zhejiang Provincial Medical and Healthy Science Foundation of China (No. 2019ZD028 and 2018KY874).

## Conflict of Interest

The authors declare that the research was conducted in the absence of any commercial or financial relationships that could be construed as a potential conflict of interest.

## References

[1] W. H. Organization Skin cancers. (2020). Retrieved from https://www.who.int/uv/faq/skincancer/en/index1.html

[2] Miller AJ Mihm MC Jr. Melanoma. N Engl J Med (2006) 355(1):51–65. 10.1056/NEJMra052166 16822996

[3] Siegel RL Miller KD Jemal A Cancer statistics, 2018. CA Cancer J Clin (2018) 68(1):7–30. 10.3322/caac.21442 29313949

[4] Furue M Kadono T Melanoma therapy: Check the checkpoints. J Dermatol (2016) 43(2):121–4. 10.1111/1346-8138.13257 26813076

[5] Sullivan RJ Atkins MB Kirkwood JM Agarwala SS Clark J II Ernstoff MS An update on the Society for Immunotherapy of Cancer consensus statement on tumor immunotherapy for the treatment of cutaneous melanoma: version 2.0. J Immunother Cancer (2018) 6(1):44. 10.1186/s40425-018-0362-6 29848375 PMC5977556

[6] Yamazaki N Uhara H Wada H Matsuda K Yamamoto K Shimamoto T Phase I study of pegylated interferon-alpha-2b as an adjuvant therapy in Japanese patients with malignant melanoma. J Dermatol (2016) 43(10):1146–53. 10.1111/1346-8138.13338 PMC5108434 27087489

[7] Kirkwood JM Ibrahim JG Sondak VK Richards J Flaherty LE Ernstoff MS High- and low-dose interferon alfa-2b in high-risk melanoma: first analysis of intergroup trial E1690/S9111/C9190. J Clin Oncol (2000) 18(12):2444–58. 10.1200/jco.2000.18.12.2444 10856105

[8] Peggs KS Quezada SA Korman AJ Allison JP Principles and use of anti-CTLA-4 antibody in human cancer immunotherapy. Curr Opin Immunol (2006) 18(2):206–13. 10.1016/j.coi.2006.01.011 16464564

[9] Sharpe AH Wherry EJ Ahmed R Freeman GJ The function of programmed cell death 1 and its ligands in regulating autoimmunity and infection. Nat Immunol (2007) 8(3):239–45. 10.1038/ni1443 17304234

[10] Cirenajwis H Ekedahl H Lauss M Harbst K Carneiro A Enoksson J Molecular stratification of metastatic melanoma using gene expression profiling: Prediction of survival outcome and benefit from molecular targeted therapy. Oncotarget (2015) 6(14):12297–309. 10.18632/oncotarget.3655 PMC4494939 25909218

[11] Perez-Guijarro E Yang HH Araya RE El Meskini R Michael HT Vodnala SK Multimodel preclinical platform predicts clinical response of melanoma to immunotherapy. Nat Med (2020) 26(5):781–91. 10.1038/s41591-020-0818-3 PMC8482620 32284588

[12] Osella-Abate S Ribero S Sanlorenzo M Maule MM Richiardi L Merletti F Risk factors related to late metastases in 1,372 melanoma patients disease free more than 10 years. Int J Cancer (2015) 136(10):2453–7. 10.1002/ijc.29281 25331444

[13] Ribero S Moscarella E Ferrara G Piana S Argenziano G Longo C Regression in cutaneous melanoma: a comprehensive review from diagnosis to prognosis. J Eur Acad Dermatol Venereol (2016) 30(12):2030–7. 10.1111/jdv.13815 27401335

[14] Tomczak K Czerwinska P Wiznerowicz M The Cancer Genome Atlas (TCGA): an immeasurable source of knowledge. Contemp Oncol (Pozn) (2015) 19(1a):A68–77. 10.5114/wo.2014.47136 PMC4322527 25691825

[15] Bhattacharya S Andorf S Gomes L Dunn P Schaefer H Pontius J ImmPort: disseminating data to the public for the future of immunology. Immunol Res (2014) 58(2-3):234–9. 10.1007/s12026-014-8516-1 24791905

[16] Edgar R Domrachev M Lash AE Gene Expression Omnibus: NCBI gene expression and hybridization array data repository. Nucleic Acids Res (2002) 30(1):207–10. 10.1093/nar/30.1.207 PMC99122 11752295

[17] Zhao K Xu L Li F Ao J Jiang G Shi R Identification of hepatocellular carcinoma prognostic markers based on 10-immune gene signature. Biosci Rep (2020) 40(8):BSR20200894. 10.1042/bsr20200894 32789471 PMC7457228

[18] Liu J Mei J Li S Wu Z Zhang Y Establishment of a novel cell cycle-related prognostic signature predicting prognosis in patients with endometrial cancer. Cancer Cell Int (2020) 20:329. 10.1186/s12935-020-01428-z 32699528 PMC7372883

[19] Newman AM Liu CL Green MR Gentles AJ Feng W Xu Y Robust enumeration of cell subsets from tissue expression profiles. Nat Methods (2015) 12(5):453–7. 10.1038/nmeth.3337 PMC4739640 25822800

[20] Subramanian A Tamayo P Mootha VK Mukherjee S Ebert BL Gillette MA Gene set enrichment analysis: a knowledge-based approach for interpreting genome-wide expression profiles. Proc Natl Acad Sci U.S.A. (2005) 102(43):15545–50. 10.1073/pnas.0506580102 PMC1239896 16199517

[21] Engebretsen S Bohlin J Statistical predictions with glmnet. Clin Epigenet (2019) 11(1):123. 10.1186/s13148-019-0730-1 PMC6708235 31443682

[22] Ritchie ME Phipson B Wu D Hu Y Law CW Shi W limma powers differential expression analyses for RNA-sequencing and microarray studies. Nucleic Acids Res (2015) 43(7):e47. 10.1093/nar/gkv007 25605792 PMC4402510

[23] Yoshihara K Shahmoradgoli M Martinez E Vegesna R Kim H Torres-Garcia W Inferring tumour purity and stromal and immune cell admixture from expression data. Nat Commun (2013) 4:2612. 10.1038/ncomms3612 24113773 PMC3826632

[24] Pencina MJ D’Agostino RB D’Agostino RB Jr. Vasan RS Evaluating the added predictive ability of a new marker: from area under the ROC curve to reclassification and beyond. Stat Med (2008) 27(2):157–72; discussion 207-12. 10.1002/sim.2929 17569110

[25] Zhou M Zhang Z Bao S Hou P Yan C Su J Computational recognition of lncRNA signature of tumor-infiltrating B lymphocytes with potential implications in prognosis and immunotherapy of bladder cancer. Brief Bioinform (2020) 00(00):1–13. 10.1093/bib/bbaa047 32382761

[26] Kalaora S Lee JS Barnea E Levy R Greenberg P Alon M Immunoproteasome expression is associated with better prognosis and response to checkpoint therapies in melanoma. Nat Commun (2020) 11(1):896. 10.1038/s41467-020-14639-9 32060274 PMC7021791

[27] Field MG Decatur CL Kurtenbach S Gezgin G van der Velden PA Jager MJ PRAME as an Independent Biomarker for Metastasis in Uveal Melanoma. Clin Cancer Res (2016) 22(5):1234–42. 10.1158/1078-0432.Ccr-15-2071 PMC4780366 26933176

[28] Ho J de Moura MB Lin Y Vincent G Thorne S Duncan LM Importance of glycolysis and oxidative phosphorylation in advanced melanoma. Mol Cancer (2012) 11:76. 10.1186/1476-4598-11-76 23043612 PMC3537610

[29] Salhi A Jordan AC Bochaca II Izsak A Darvishian F Houvras Y Oxidative Phosphorylation Promotes Primary Melanoma Invasion. Am J Pathol (2020) 190(5):1108–17. 10.1016/j.ajpath.2020.01.012 PMC7237828 32142731

[30] Amann PM Luo C Owen RW Hofmann C Freudenberger M Schadendorf D Vitamin A metabolism in benign and malignant melanocytic skin cells: importance of lecithin/retinol acyltransferase and RPE65. J Cell Physiol (2012) 227(2):718–28. 10.1002/jcp.22779 21465477

[31] Hassel JC Amann PM Schadendorf D Eichmuller SB Nagler M Bazhin AV Lecithin retinol acyltransferase as a potential prognostic marker for malignant melanoma. Exp Dermatol (2013) 22(11):757–9. 10.1111/exd.12236 24433184

[32] Amann PM Czaja K Bazhin AV Ruhl R Skazik C Heise R Knockdown of lecithin retinol acyltransferase increases all-trans retinoic acid levels and restores retinoid sensitivity in malignant melanoma cells. Exp Dermatol (2014) 23(11):832–7. 10.1111/exd.12548 25236354

[33] El Hassouni B Sarkisjan D Vos JC Giovannetti E Peters GJ Targeting the Ribosome Biogenesis Key Molecule Fibrillarin to Avoid Chemoresistance. Curr Med Chem (2019) 26(33):6020–32. 10.2174/0929867326666181203133332 30501594

[34] Scull CE Zhang Y Tower N Rasmussen L Padmalayam I Hunter R Discovery of novel inhibitors of ribosome biogenesis by innovative high throughput screening strategies. Biochem J (2019) 476(15):2209–19. 10.1042/bcj20190207 PMC7278283 31341008

[35] Xiong J Bing Z Guo S Observed Survival Interval: A Supplement to TCGA Pan-Cancer Clinical Data Resource. Cancers (Basel) (2019) 11(3):280. 10.3390/cancers11030280 PMC6468755 30813652

[36] Human genomics The Genotype-Tissue Expression (GTEx) pilot analysis: multitissue gene regulation in humans. Science (2015) 348(6235):648–60. 10.1126/science.1262110 PMC4547484 25954001

[37] Girotti MR Saturno G Lorigan P Marais R No longer an untreatable disease: how targeted and immunotherapies have changed the management of melanoma patients. Mol Oncol (2014) 8(6):1140–58. 10.1016/j.molonc.2014.07.027 PMC5528622 25178978

[38] Hoag H Drug development: a chance of survival. Nature (2014) 515(7527):S118–20. 10.1038/515S118a 25407709

[39] Hodi FS Chiarion-Sileni V Gonzalez R Grob JJ Rutkowski P Cowey CL Nivolumab plus ipilimumab or nivolumab alone versus ipilimumab alone in advanced melanoma (CheckMate 067): 4-year outcomes of a multicentre, randomised, phase 3 trial. Lancet Oncol (2018) 19(11):1480–92. 10.1016/s1470-2045(18)30700-9 30361170

[40] Schadendorf D Hodi FS Robert C Weber JS Margolin K Hamid O Pooled Analysis of Long-Term Survival Data From Phase II and Phase III Trials of Ipilimumab in Unresectable or Metastatic Melanoma. J Clin Oncol (2015) 33(17):1889–94. 10.1200/jco.2014.56.2736 PMC5089162 25667295

[41] Kaufman HL Kirkwood JM Hodi FS Agarwala S Amatruda T Bines SD The Society for Immunotherapy of Cancer consensus statement on tumour immunotherapy for the treatment of cutaneous melanoma. Nat Rev Clin Oncol (2013) 10(10):588–98. 10.1038/nrclinonc.2013.153 23982524

[42] Subrahmanyam PB Dong Z Gusenleitner D Giobbie-Hurder A Severgnini M Zhou J Distinct predictive biomarker candidates for response to anti-CTLA-4 and anti-PD-1 immunotherapy in melanoma patients. J Immunother Cancer (2018) 6(1):18. 10.1186/s40425-018-0328-8 29510697 PMC5840795

[43] Guo W Zhu L Zhu R Chen Q Wang Q Chen JQ A four-DNA methylation biomarker is a superior predictor of survival of patients with cutaneous melanoma. Elife (2019) 8:e44310. 10.7554/eLife.44310 31169496 PMC6553943

[44] Li B Cui Y Diehn M Li R Development and Validation of an Individualized Immune Prognostic Signature in Early-Stage Nonsquamous Non-Small Cell Lung Cancer. JAMA Oncol (2017) 3(11):1529–37. 10.1001/jamaoncol.2017.1609 PMC5710196 28687838

[45] Zhang L Zhu P Tong Y Wang Y Ma H Xia X An immune-related gene pairs signature predicts overall survival in serous ovarian carcinoma. Onco Targets Ther (2019) 12:7005–14. 10.2147/ott.S200191 PMC6718165 31695415

[46] Emran AA Nsengimana J Punnia-Moorthy G Schmitz U Gallagher SJ Newton-Bishop J Study of the Female Sex Survival Advantage in Melanoma-A Focus on X-Linked Epigenetic Regulators and Immune Responses in Two Cohorts. Cancers (Basel) (2020) 12(8):2082. 10.3390/cancers12082082 PMC7464825 32731355

[47] Cho JH Robinson JP Arave RA Burnett WJ Kircher DA Chen G AKT1 Activation Promotes Development of Melanoma Metastases. Cell Rep (2015) 13(5):898–905. 10.1016/j.celrep.2015.09.057 26565903 PMC4646731

[48] Weide B Allgaier N Hector A Forschner A Leiter U Eigentler TK Increased CCL17 serum levels are associated with improved survival in advanced melanoma. Cancer Immunol Immunother (2015) 64(9):1075–82. 10.1007/s00262-015-1714-4 PMC11029296 25990074

[49] Singh M Vianden C Cantwell MJ Dai Z Xiao Z Sharma M Intratumoral CD40 activation and checkpoint blockade induces T cell-mediated eradication of melanoma in the brain. Nat Commun (2017) 8(1):1447. 10.1038/s41467-017-01572-7 29129918 PMC5682289

[50] Smith MP Rowling EJ Miskolczi Z Ferguson J Spoerri L Haass NK Targeting endothelin receptor signalling overcomes heterogeneity driven therapy failure. EMBO Mol Med (2017) 9(8):1011–29. 10.15252/emmm.201607156 PMC5538298 28606996

[51] Sangalli A Orlandi E Poli A Maurichi A Santinami M Nicolis M Sex-specific effect of RNASEL rs486907 and miR-146a rs2910164 polymorphisms’ interaction as a susceptibility factor for melanoma skin cancer. Melanoma Res (2017) 27(4):309–14. 10.1097/cmr.0000000000000360 28654546

[52] Ma B Herzog EL Lee CG Peng X Lee CM Chen X Role of chitinase 3-like-1 and semaphorin 7a in pulmonary melanoma metastasis. Cancer Res (2015) 75(3):487–96. 10.1158/0008-5472.Can-13-3339 PMC4321965 25511377

[53] Lopez-Janeiro A Padilla-Ansala C de Andrea CE Hardisson D Melero I Prognostic value of macrophage polarization markers in epithelial neoplasms and melanoma. A systematic review and meta-analysis. Mod Pathol (2020) 33(8):1458–65. 10.1038/s41379-020-0534-z 32291396

[54] Yamaguchi K Mishima K Ohmura H Hanamura F Ito M Nakano M Activation of central/effector memory T cells and T-helper 1 polarization in malignant melanoma patients treated with anti-programmed death-1 antibody. Cancer Sci (2018) 109(10):3032–42. 10.1111/cas.13758 PMC6172076 30066977

[55] Xue Y Xue Y Wang Z Mo Y Wang P Tan W A Novel Signature of 23 Immunity-related Gene Pairs is Prognostic of Cutaneous Melanoma. PREPRINT. 10.21203/rs.3.rs-38060/v1. (Posted 29 Jun, 2020) available at Rsearch Square. PMC7604355 33193373

