# Supplementary material for: A Novel Signature of 23 Immunity-Related Gene Pairs Is Prognostic of Cutaneous Melanoma
Source: Front Immunol. 2020 Oct 19;11:576914. doi: 10.3389/fimmu.2020.576914 (PMC7604355; doi:10.3389/fimmu.2020.576914)
Supplement: Supplementary file 1 [file Table_1.docx]

**Table S1.** 16 GO terms enriched in the high risk group

| Term | pval | padj | ES | NES | size |
| --- | --- | --- | --- | --- | --- |
| KERATINIZATION | 0.001043841 | 0.01578043 | 0.885403386 | 3.061054102 | 223 |
| EPIDERMAL_CELL_DIFFERENTIATION | 0.001841621 | 0.02502996 | 0.829088947 | 2.997508748 | 350 |
| KERATIN_FILAMENT | 0.000528541 | 0.009898822 | 0.949239926 | 2.957232759 | 95 |
| INTERMEDIATE_FILAMENT_CYTOSKELETON | 0.001078749 | 0.016070903 | 0.843744562 | 2.948487442 | 238 |
| SKIN_DEVELOPMENT | 0.002506266 | 0.031517643 | 0.789434948 | 2.895519502 | 412 |
| EPIDERMIS_DEVELOPMENT | 0.003205128 | 0.036939983 | 0.782459 | 2.867529739 | 457 |
| CORNIFICATION | 0.000586166 | 0.01037523 | 0.859956033 | 2.744762952 | 112 |
| CORNIFIED_ENVELOPE | 0.000444444 | 0.008506083 | 0.896979351 | 2.62500738 | 65 |
| PEPTIDE_CROSS_LINKING | 0.000428082 | 0.008222941 | 0.790742331 | 2.292802166 | 60 |
| REGULATION_OF_WATER_LOSS_VIA_SKIN | 0.00034638 | 0.007151254 | 0.857145769 | 2.073693614 | 24 |
| STRUCTURAL_CONSTITUENT_OF_EPIDERMIS | 0.00032175 | 0.006776139 | 0.916475766 | 2.043453586 | 16 |
| DESMOSOME | 0.001044205 | 0.01578043 | 0.791252315 | 1.933833569 | 25 |
| INTERMEDIATE_FILAMENT_BASED_PROCESS | 0.000786164 | 0.012910805 | 0.693316224 | 1.929332376 | 48 |
| MOLTING_CYCLE | 0.000580383 | 0.010317047 | 0.577901789 | 1.842938547 | 111 |
| STRUCTURAL_CONSTITUENT_OF_CYTOSKELETON | 0.000535906 | 0.009984829 | 0.552345522 | 1.728494398 | 98 |
| UNSATURATED_FATTY_ACID_METABOLIC_PROCESS | 0.002816901 | 0.033958232 | 0.543223201 | 1.639773602 | 76 |

**Table S2.** Clinical and pathologic factors of the TCGA datasets used in this study.

|  | TCGA-train dataset (n = 230) | TCGA-test dataset (n = 230) | | TCGA dataset (n = 460) | GSE65904 (n = 214) | GSE59455 (n = 141) | GSE22153 (n = 79) |
| --- | --- | --- | --- | --- | --- | --- | --- |
| Age | | | | |  |  |  |
| ≤ 65 | 113(49.1%) | | 129(56.1%) | 242(62.2%) | 113(52.8%) | 51(36.2%) | 39(49.4%) |
| ＞ 65 | 117(50.9%) | | 101(43.9%) | 218(27.8%) | 90(42.1%) | 72(51.1%) | 18(22.8%) |
| Unknown | 0 | | 0 | 0 | 11(5.1%) | 18(12.7%) | 22(27.8%) |
| Gender | | | | |  |  |  |
| Male | 144(62.6%) | 142(61.7%) | | 286(62.4%) | 119(55.6%) | 69(48.9%) | 31(39.2%) |
| Female | 86(37.4%) | 88(38.3%) | | 174(37.6%) | 85(39.7%) | 45(31.9%) | 26(33.0%) |
| Unknown | 0 | 0 | | 0 | 10(4.7%) | 27(19.1%) | 22(27.8%) |
| Pathologic stage | | | | |  |  |  |
| 0 | 4(1.7%) | 2(0.9%) | | 6(1.3%) | 0 | 0 | 0 |
| I-II | 110(47.8%) | 104(45.2%) | | 214(46.5%) | 27(12.6%) | 39(27.7%) | 0 |
| III-IV | 95(41.3%) | 99(43.0%) | | 194(42.2%) | 177(82.7%) | 84(59.6%) | 57(72.2%) |
| Unknown | 21(9.2%) | 25(10.9%) | | 46(10.0%) | 10(4.7%) | 18(12.7%) | 22(27.8%) |
| Survival status | | | | |  |  |  |
| Dead | 112(46.7%) | 128(55.7%) | | 240(52.2%) | 102(47.7%) | 123(87.2%) | 47(59.5%) |
| Alive | 118(53.3%) | 102(44.3%) | | 220(47.8%) | 108(50.5%) | 0 | 7(8.9%) |
| Unknown | 0 | 0 | | 0 | 4(1.8%) | 18(12.8%) | 25(31.6%) |

**Table S3.** 1-, 3-, 5-year AUC values of different datasets.

| Dataset | AUC(1-year) | AUC(3-year) | AUC(5-year) |
| --- | --- | --- | --- |
| TCGA-train dataset | 0.909 | 0.901 | 0.912 |
| TCGA-test dataset | 0.927 | 0.902 | 0.937 |
| TCGA dataset | 0.913 | 0.901 | 0.926 |
| IRGs | 0.731 | 0.76 | 0.749 |
| IRGP-OBS | 0.946 | 0.928 | 0.957 |
