# Supplementary material for: A Novel Signature of 23 Immunity-Related Gene Pairs Is Prognostic of Cutaneous Melanoma
Source: Front Immunol. 2020 Oct 19;11:576914. doi: 10.3389/fimmu.2020.576914 (PMC7604355; doi:10.3389/fimmu.2020.576914)
Supplement: Supplementary file 2 [file Table_2.docx]

Supplementary Figures

**
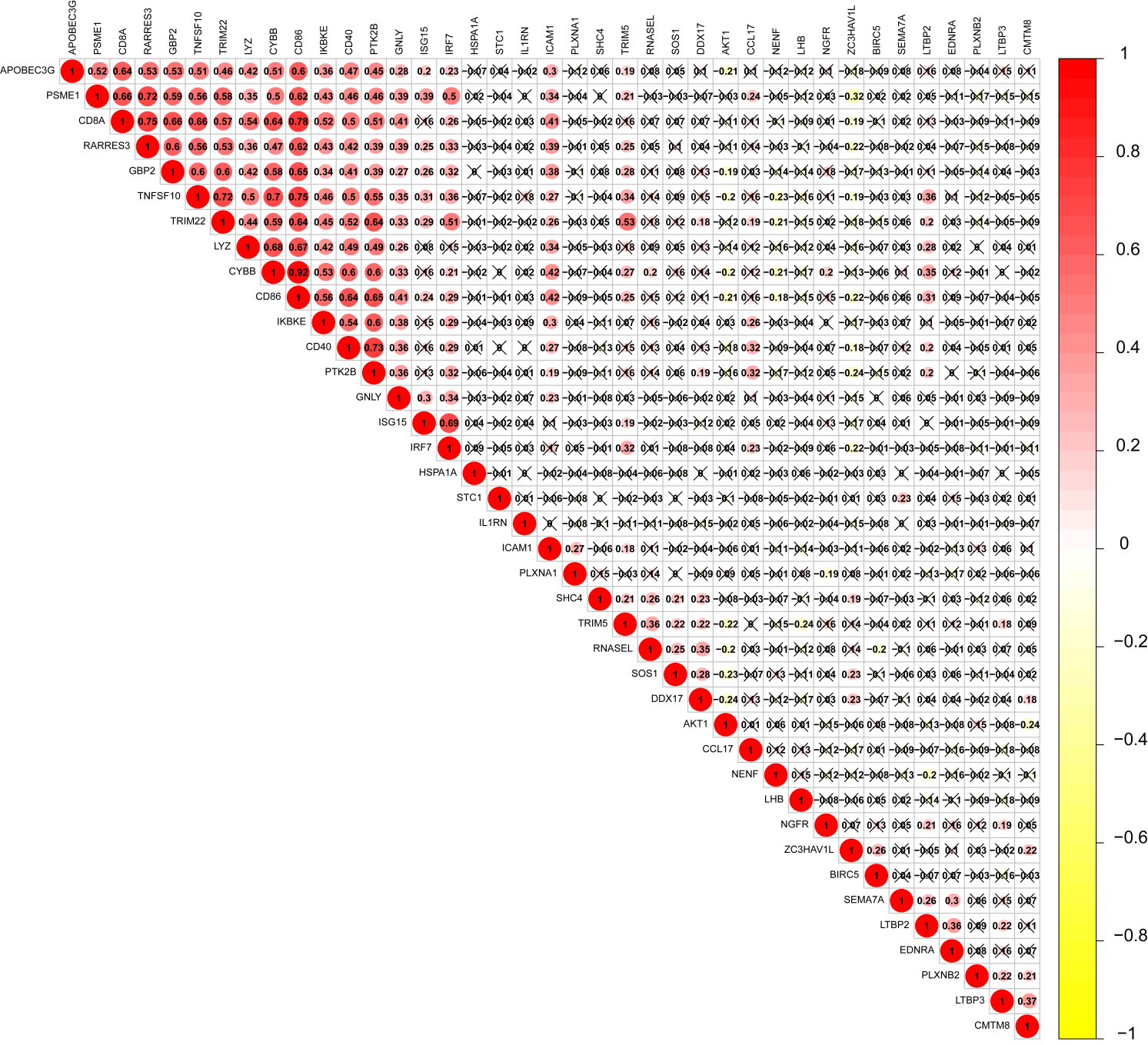
**

**Figure S1** The mutation co-occurrence and exclusion analyses for 39 IRGs. Co-occurrence, red; Exclusion, yellow.

**
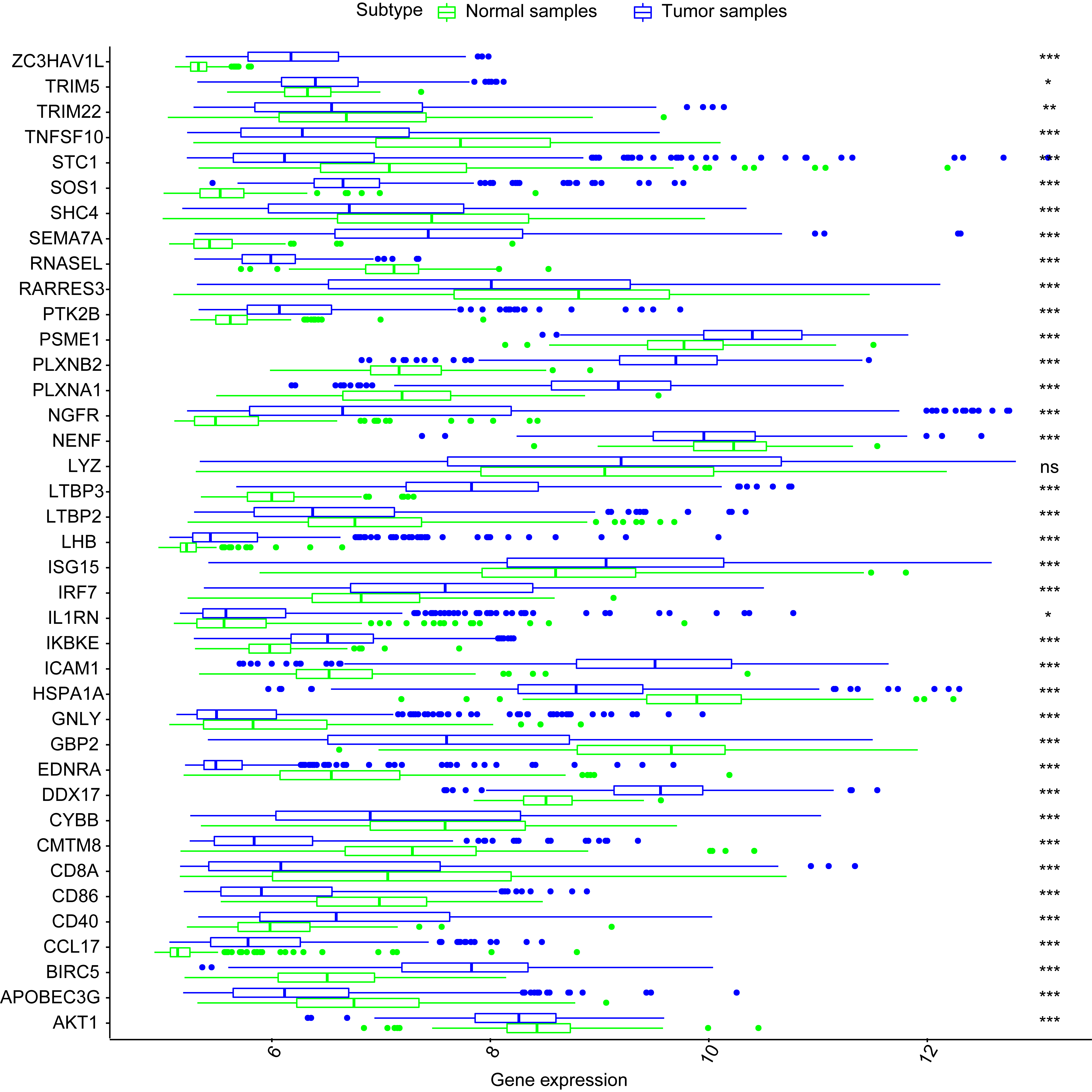
**

**Figure S2**  The expression of 39 IRGs between tumor samples and normal samples. Tumor, blue; Normal, green. The lines in the boxes represented median value, the upper and lower ends of the boxes represented interquartile range of values, and the dots showed outliers. The asterisks represented the statistical p value (*P< 0.05; **P< 0.01; ***P< 0.001).


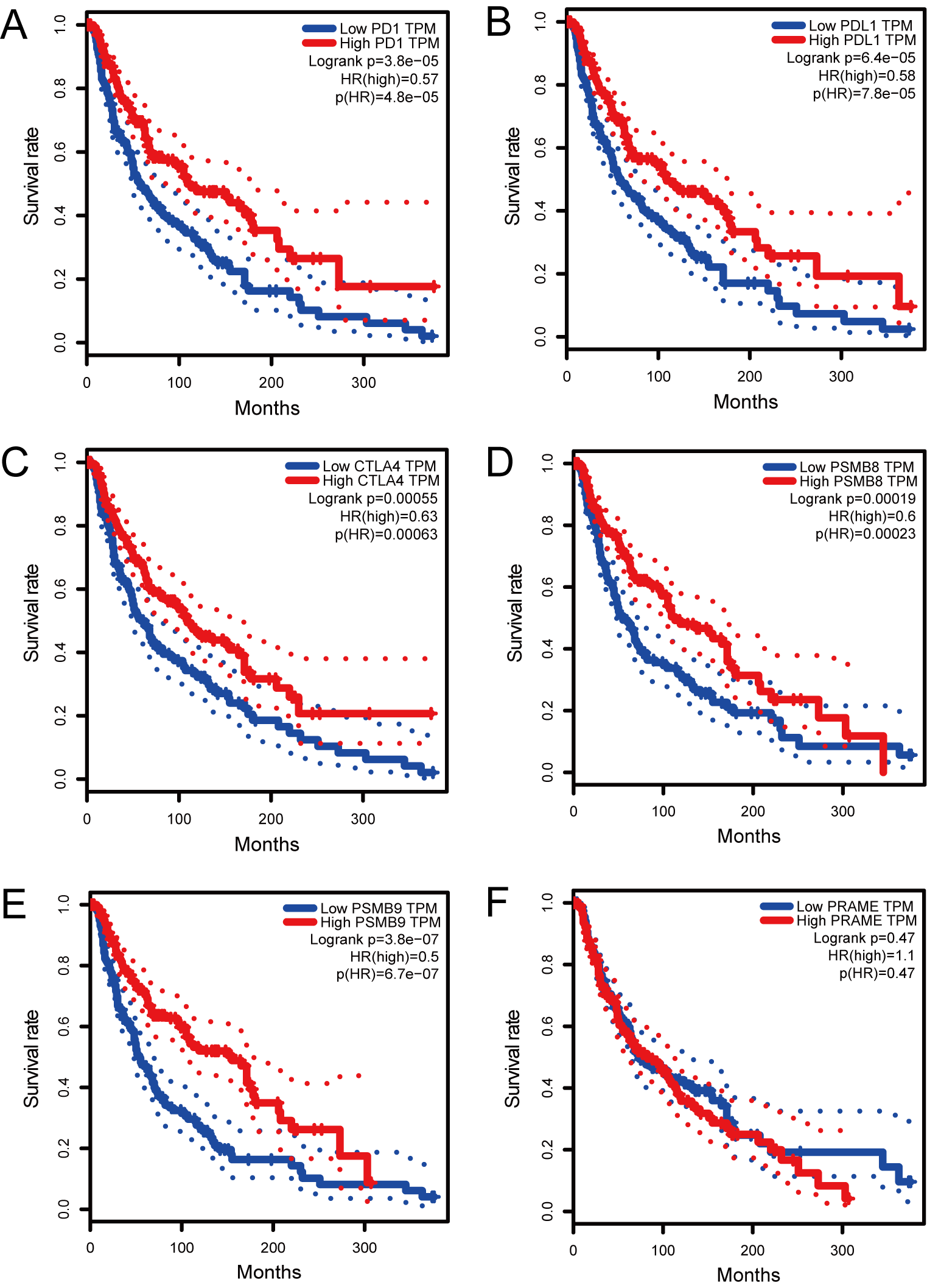


**Figure S3** In GEPIA, the patients with high PD1, PDL-1, CTLA4, PSMB8 and PSMB9 expression had better OS (A-E), but PRAME had no significant effect in OS (F).


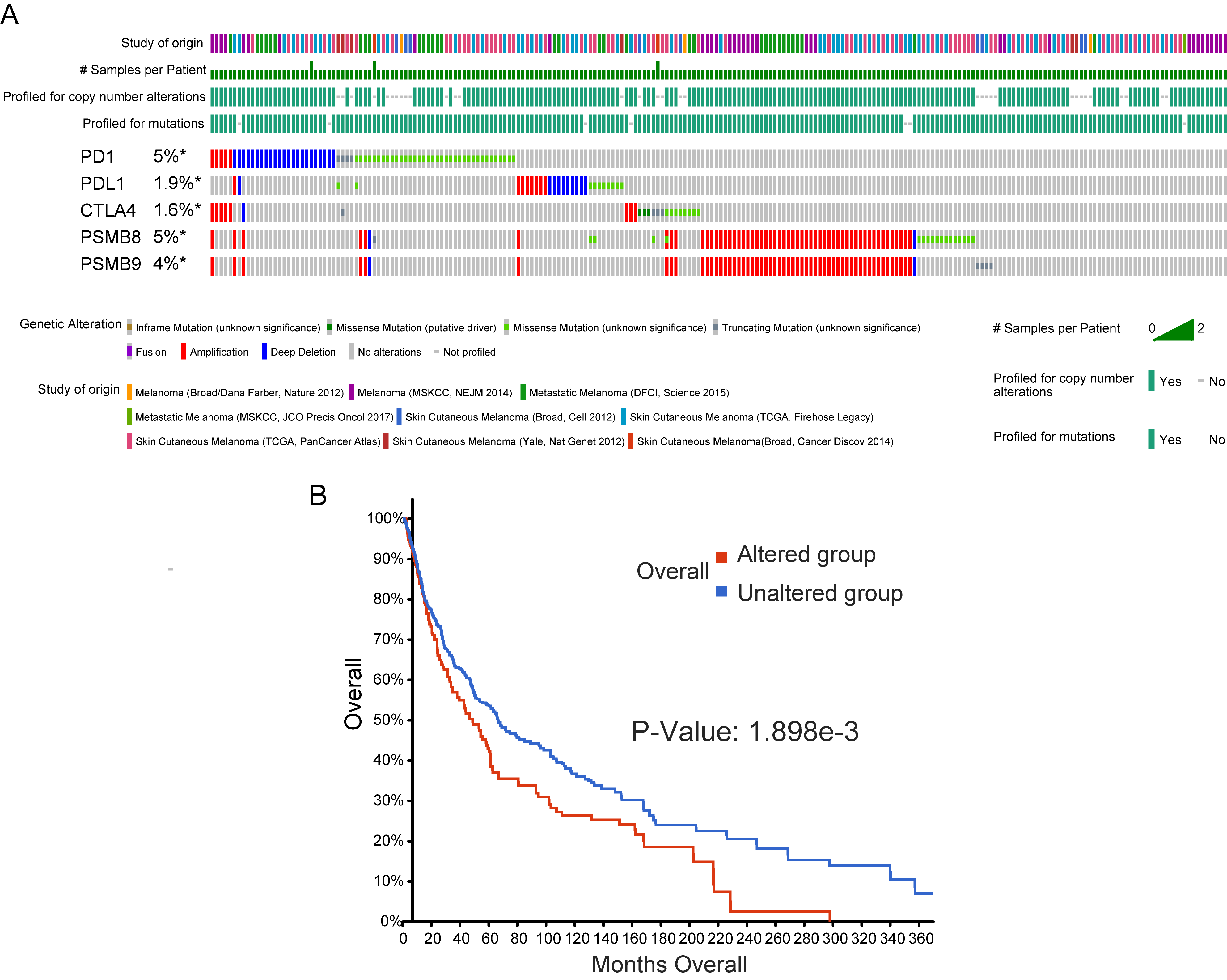


**Figure S4** Nine melanoma datasets (n = 1539) were used to explored the mutation of 5 key genes that highly expressed in low immune group. PD1, PDL-1, CTLA4, PSMB8 and PSMB9 had 5%, 1.9%, 1.6%, 5% and 4% mutation probability. Poorer OS occured if these genes altered.

**
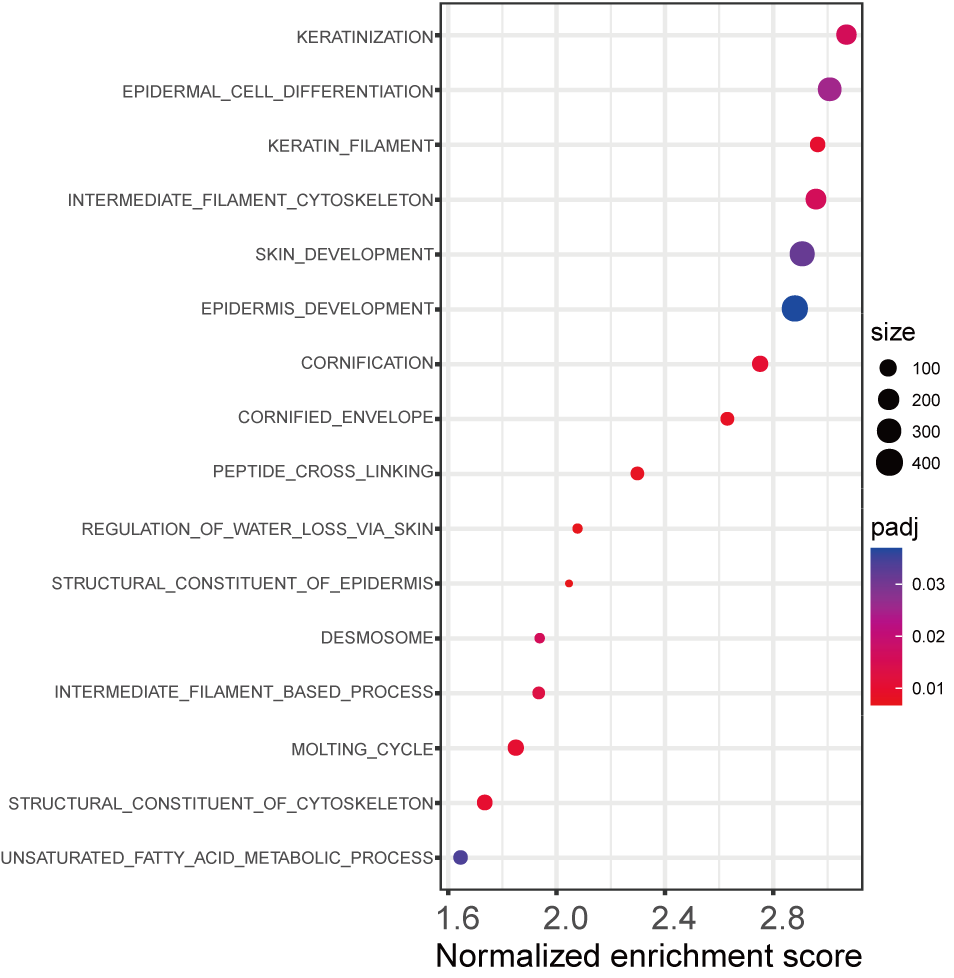
**

**Figure S5** GO analysis of the high-immune risk group based on the 23 IRGPs (padj < 0.05). “Normalized enrichment score” is the percentage of differentiated genes for the given GO term.

**
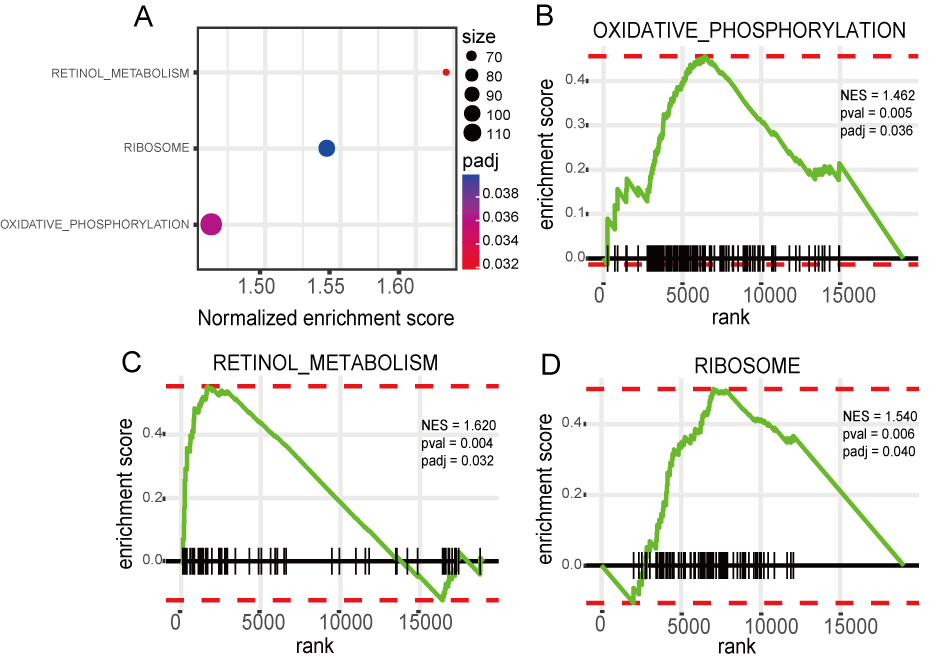
**

**Figure S6** KEGG pathway analysis of the high immune risk group based on the 23 IRGPs. KEGG pathway analysis bubble plot(A) confirmed that melanoma metastasis-related pathways such as oxidative phosphorylation(B), retinol metabolism(C), and ribosome(D), were upregulated in the high risk group (padj < 0.05).


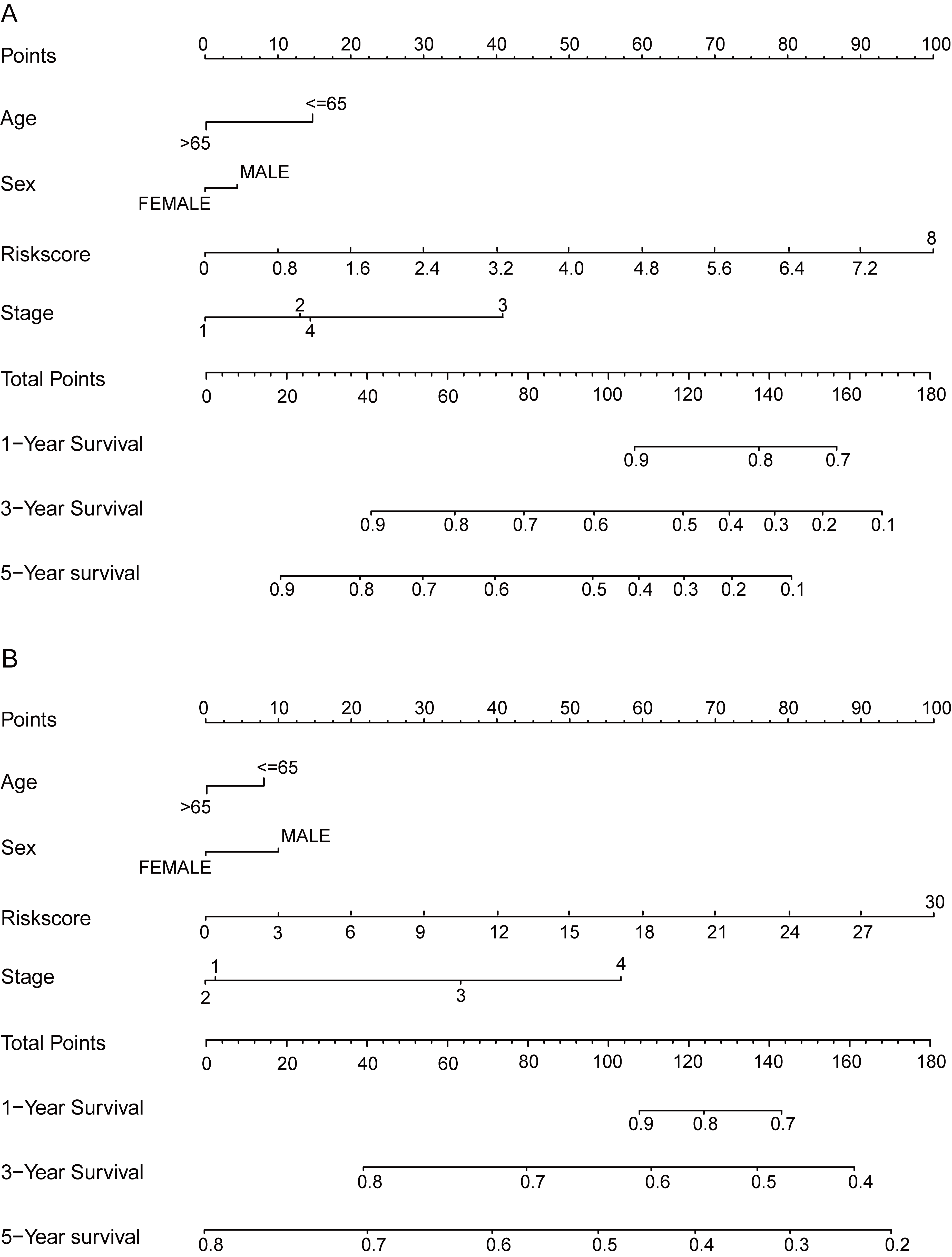


**Figure S7** The TCGA-test dataset nomogram(A) and TCGA dataset nomogram(B) of OS were established by 23-IRGP risk score and other clinicopathological parameters.


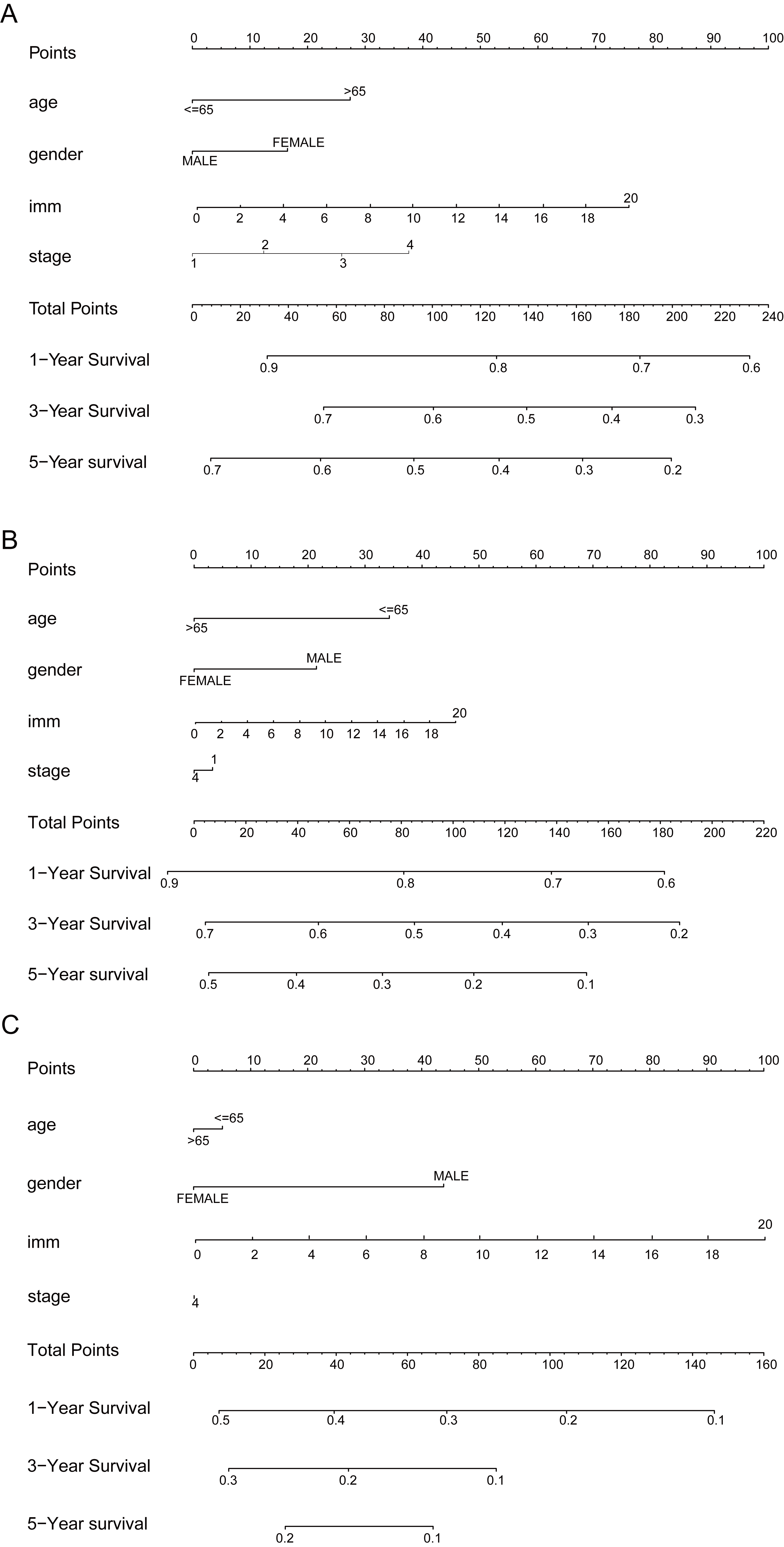


**Figure S8** The GSE65904 dataset nomogram(A), GSE59455 dataset nomogram(B) and GSE54467(C) of OS were established by 23-IRGP risk score and other clinicopathological parameters.


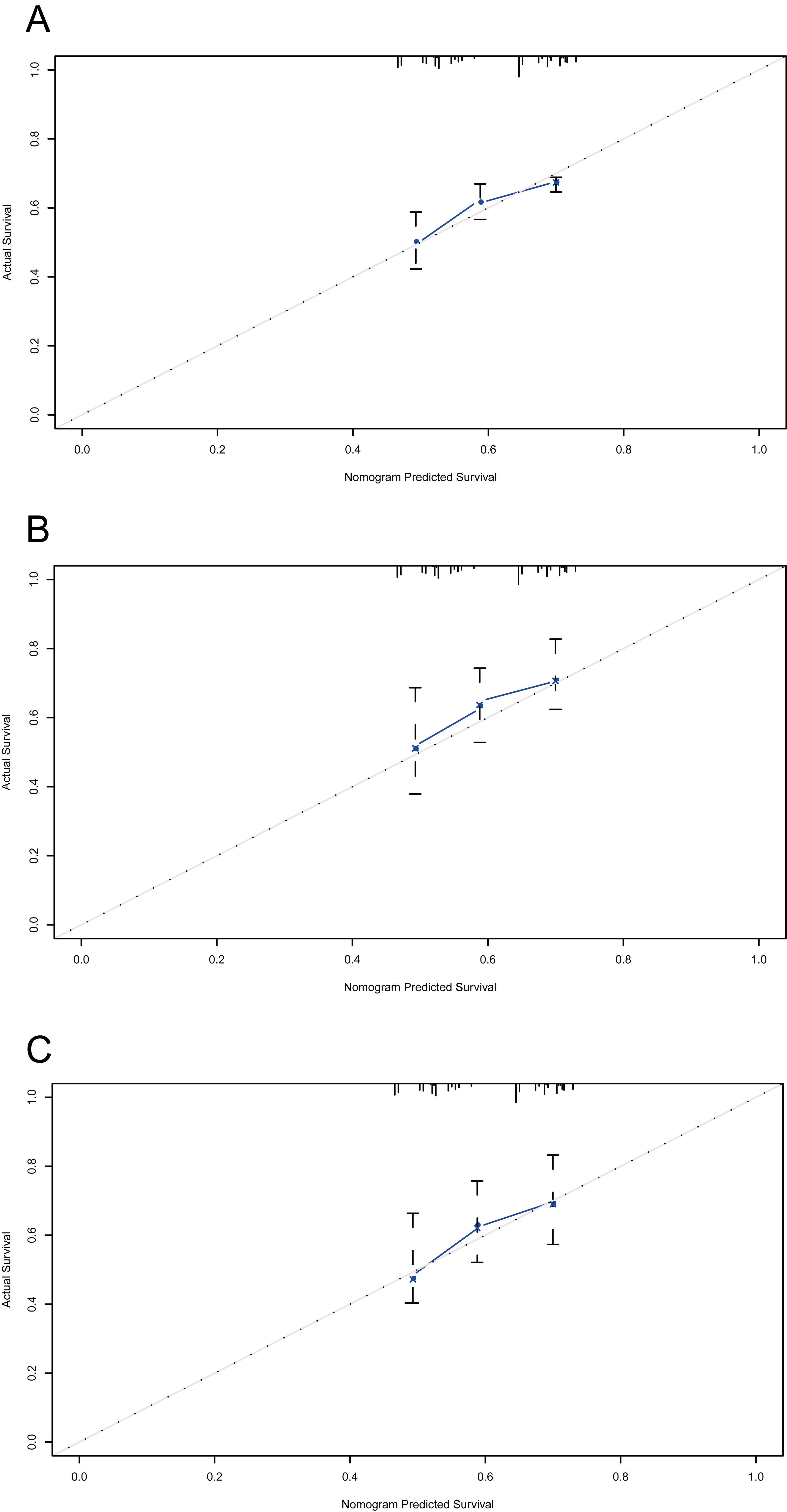


**Figure S9** The 1-(A), 3-(B), and 5-year(C) calibration curves of the TCGA-test dataset nomogram.


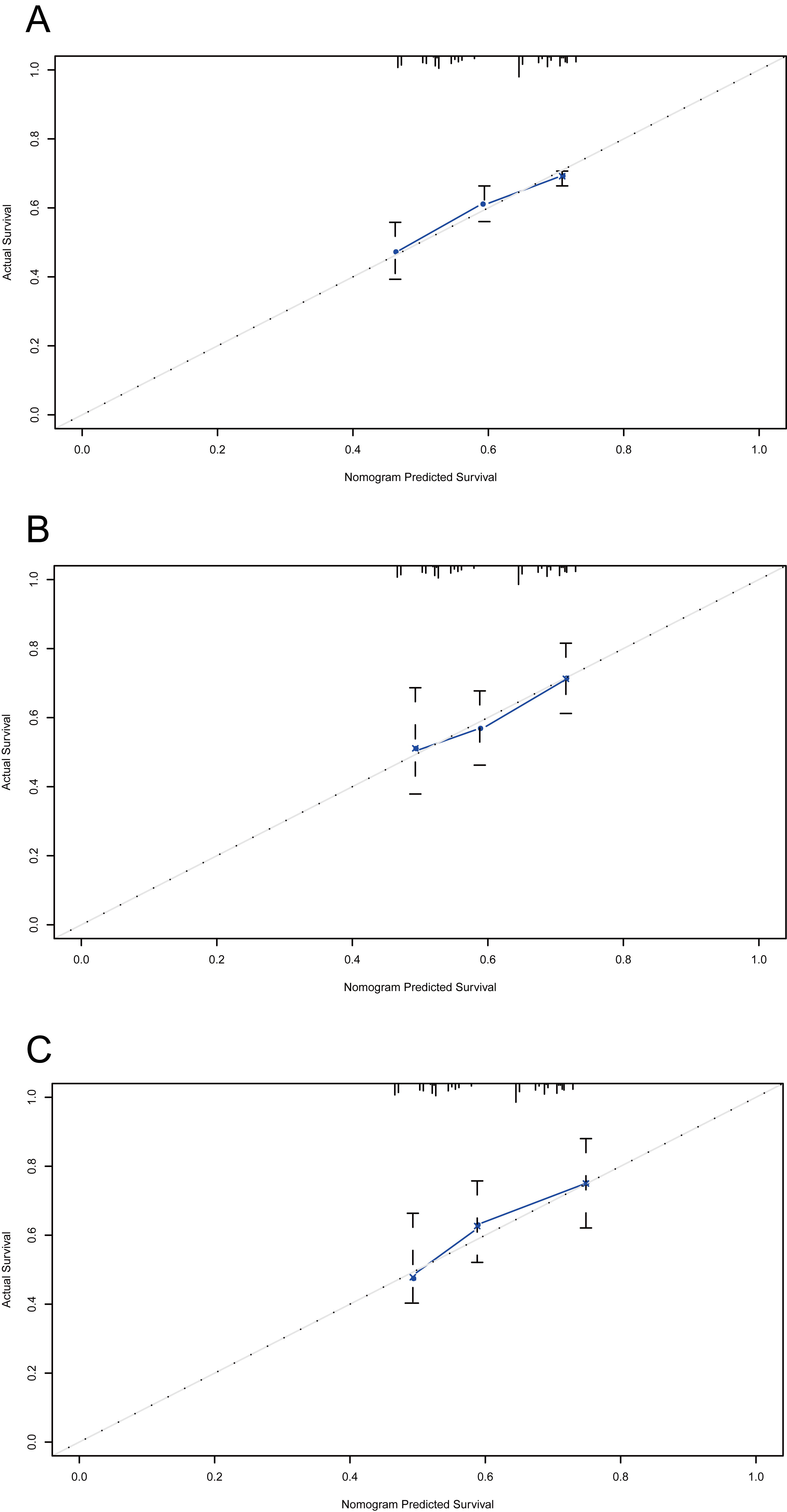


**Figure S10** The 1-(A), 3-(B), and 5-year(C) calibration curves of the TCGA dataset nomogram.


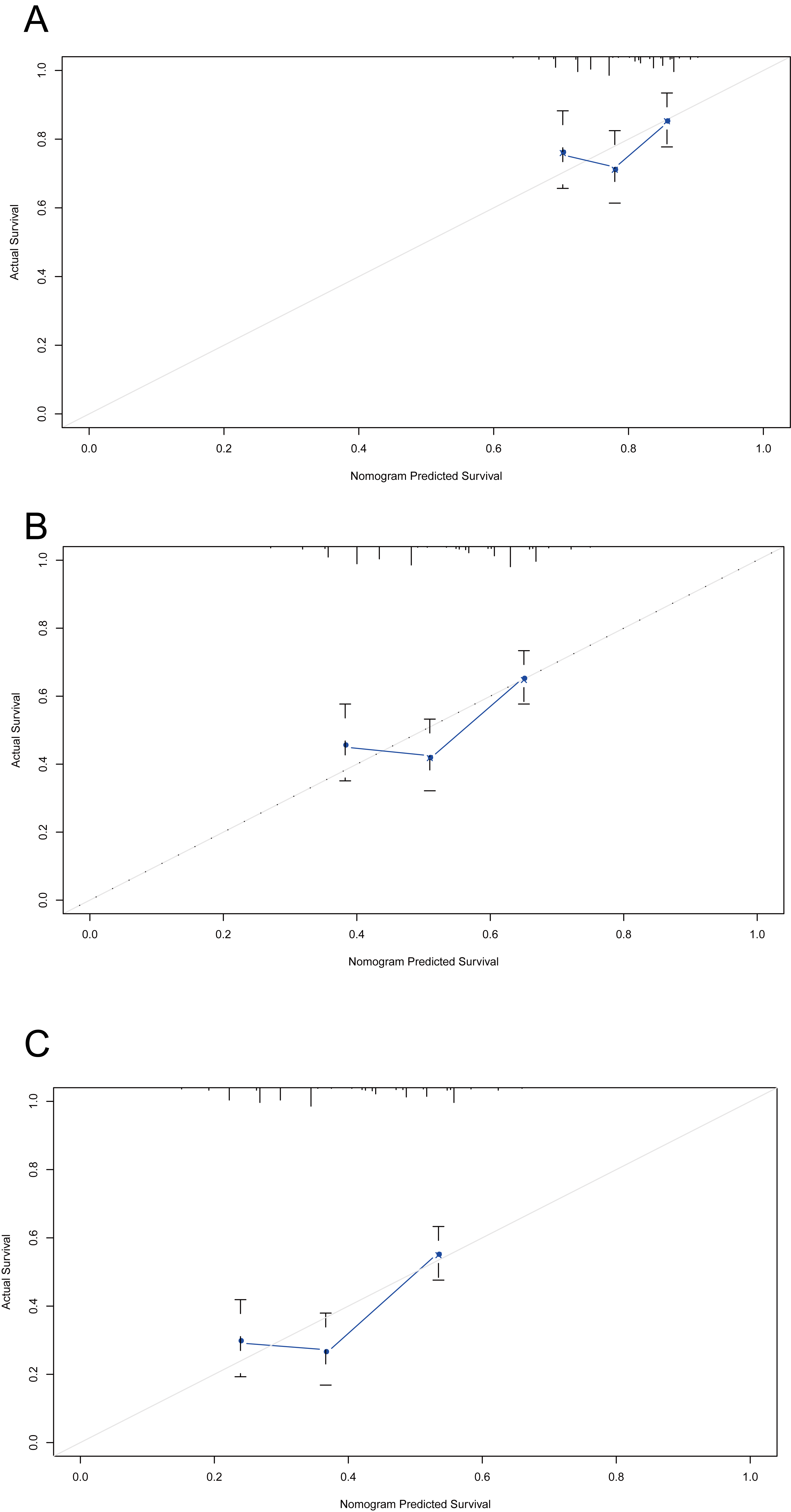


**Figure S11** The 1-(A), 3-(B), and 5-year(C) calibration curves of the GSE65904 dataset nomogram.


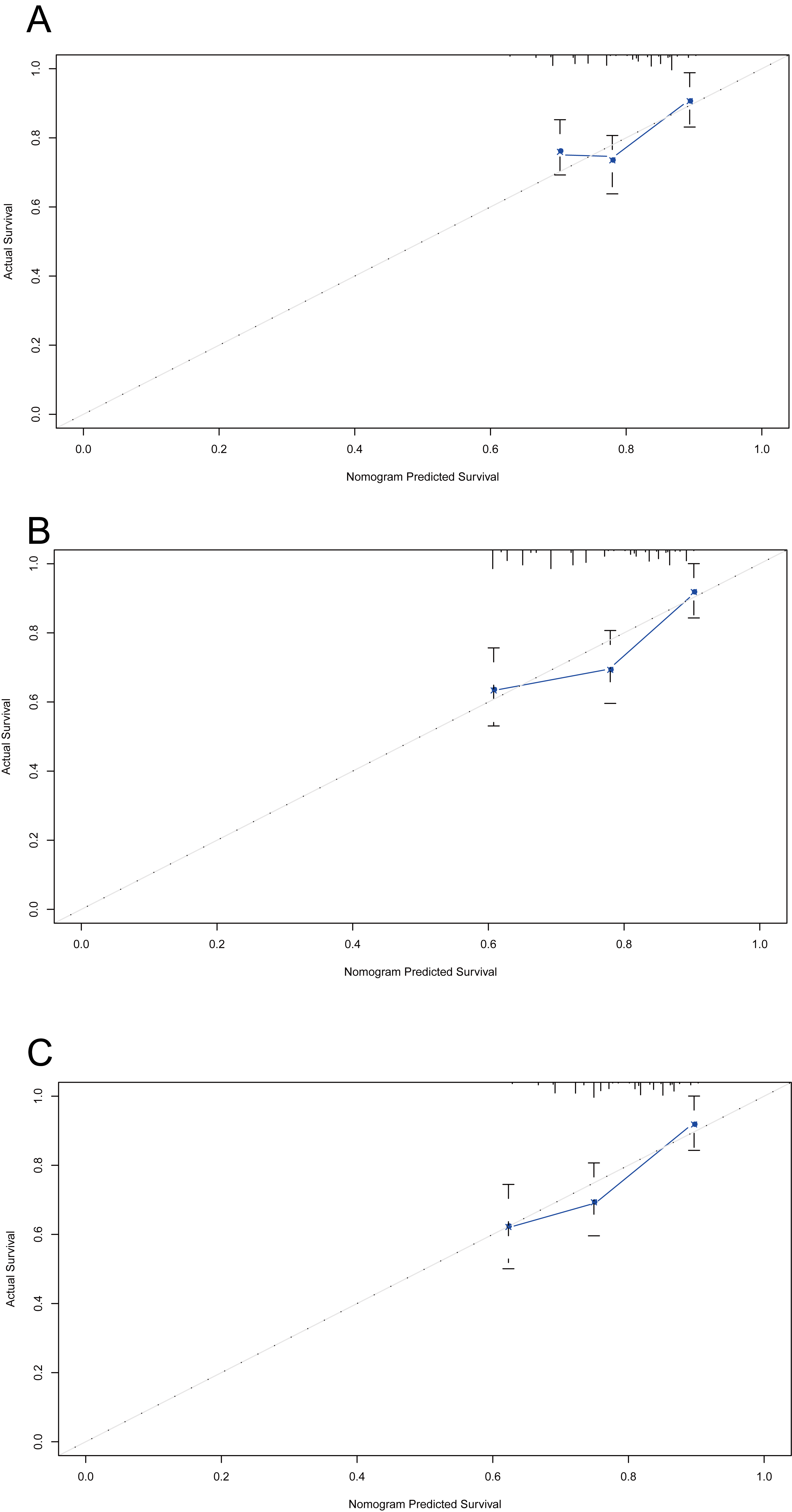


**Figure S12** The 1-(A), 3-(B), and 5-year(C) calibration curves of the GSE59455 dataset nomogram.


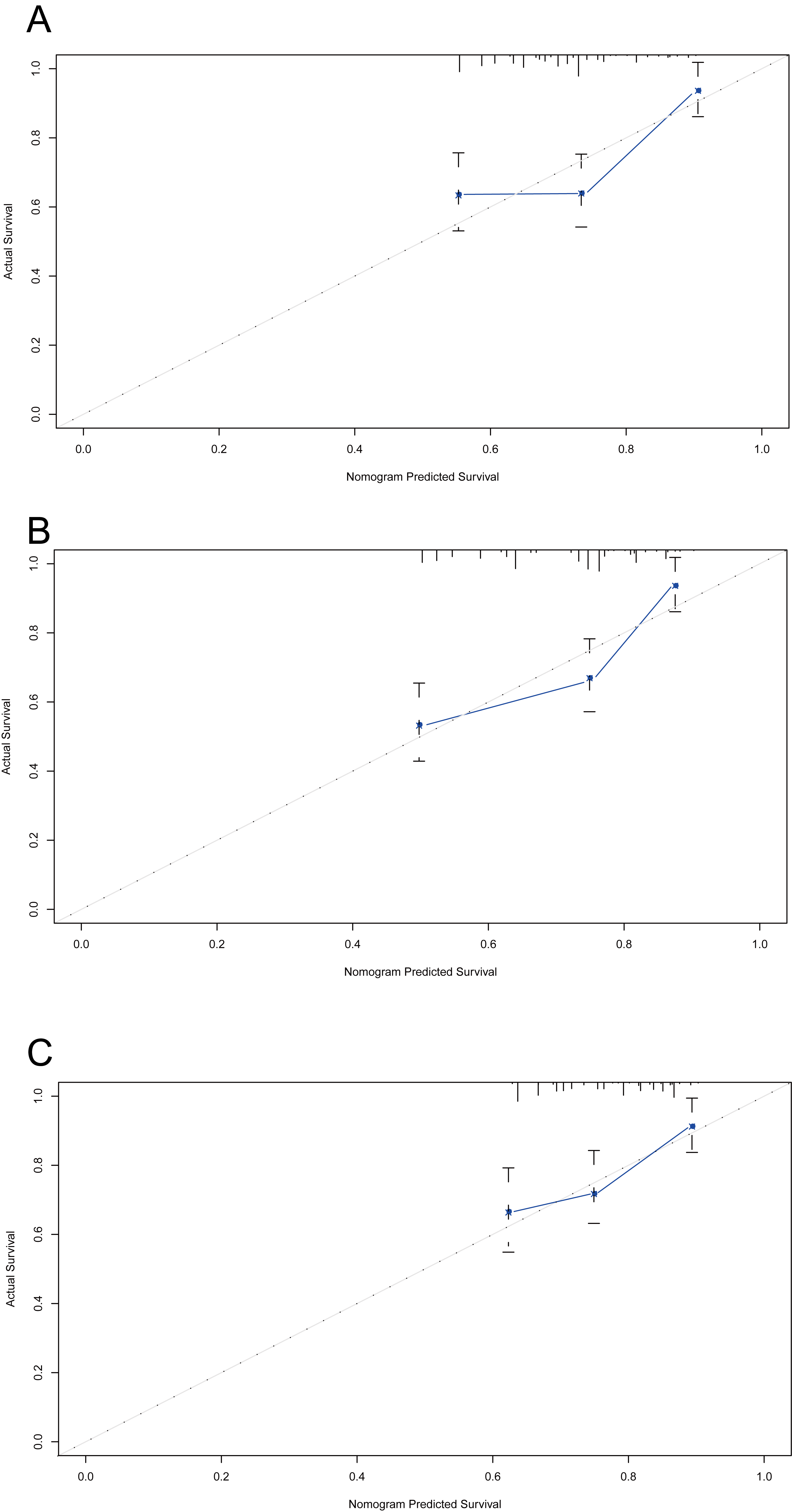


**Figure S13** The 1-(A), 3-(B), and 5-year(C) calibration curves of the GSE22153 dataset nomogram.


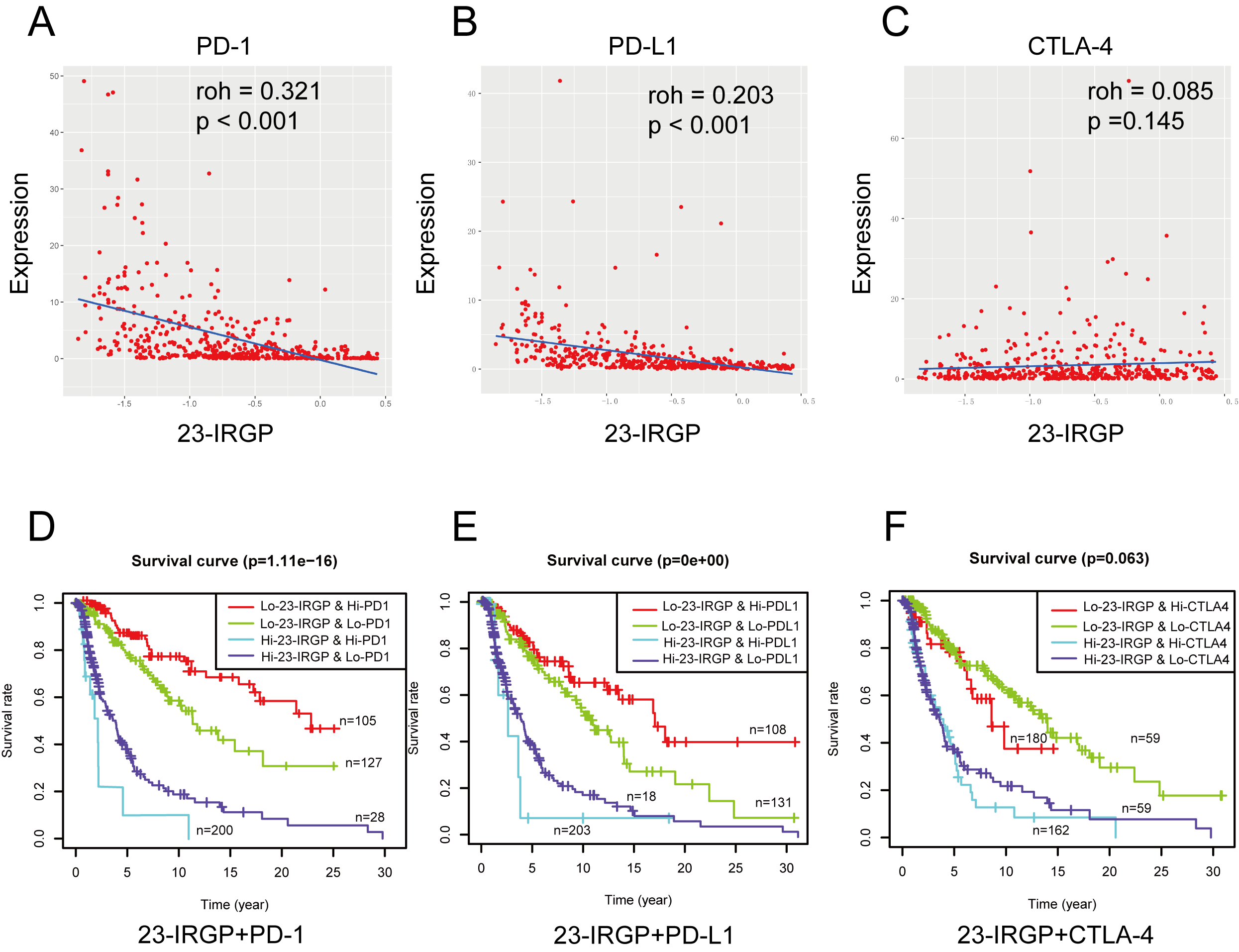


**Figure S14** Effect of the 23-IRGP and ICIs expression on CM patient survival. (A) Correlation between the 23-IRGP and PD-1 expression. (B) Correlation between the 23-IRGP and PD-L1 expression. (C) Correlation between the 23-IRGP and CTLA-4 expression. (D) Kaplan–Meier survival curves of OS among four patient clusters divided by the 23-IRGP and PD1 expression. (E) Kaplan–Meier survival curves of OS among four patient clusters divided by the 23-IRGP and PD-L1 expression. (F) Kaplan–Meier survival curves of OS among four patient clusters divided by the 23-IRGP and CTLA-4 expression.
